# Multichiral Half-Sandwich Ru(II) and Os(II) Anticancer Complexes Containing a Glutathione Synthesis Inhibitor

**DOI:** 10.1021/acs.organomet.5c00375

**Published:** 2025-12-11

**Authors:** Pragya Kumari, Hannah E. Bridgewater, Sara Anisi, Craig M. Whitehouse, Adam J. Millett, Prinessa Chellan, Isolda Romero-Canelón, Guy J. Clarkson, Volker Schünemann, Juliusz A. Wolny, Peter J. Sadler

**Affiliations:** † Department of Chemistry, 2707 University of Warwick, Gibbet Hill Road, Coventry CV4 7AL, United Kingdom; ‡ Centre for Health and Life Sciences, 2706 Coventry University, Priory Street, Coventry CV1 5FB, United Kingdom; § School of Life Sciences, 2707 University of Warwick, Gibbet Hill Road, Coventry, CV4 7AL, United Kingdom; ∥ Department of Chemistry and Polymer Science, 26697 Stellenbosch University, 7600 Matieland, Western Cape, South Africa; ⊥ School of Pharmacy, 1724 University of Birmingham, Birmingham B15 2TT, United Kingdom; # Department of Physics, 26562 RPTU University Kaiserslautern-Landau, Erwin-Schrödinger-Straße 46, 67663 Kaiserslautern, Germany

## Abstract

Two novel half-sandwich organometallic complexes, [(*p*-cymene)­M­(XY)­Cl], XY = *L*-BSO, M = Ru^II^ (**Ru-LBSO**), Os^II^ (**Os-LBSO**), containing the amino acid *L*-buthionine sulfoximine (*L*-BSO), as well as their XY = glycine analogs (**Ru-Gly** and **Os-Gly**), have been synthesized, characterized and their solution chemistry investigated. *L*-BSO is an inhibitor of the enzyme γ-glutamyl cysteine synthetase and, hence, glutathione synthesis. The diastereomers of **Ru-LBSO** and **Os-LBSO** were also characterized by DFT calculations which suggested the higher stability of [S_M_,r_S_] and [S_M_,s_S_] compared to [R_M_,r_S_] and [R_M_,s_S_] configurations [chirality at M­(II), chirality at sulfur of *L*-BSO]. Interestingly, glycine complexes are non-toxic toward both cancer and normal cells, whereas **Os-LBSO** was cytotoxic toward human IGROV-1 ovarian cancer cells, but not toward lung and cervical cancer cells. **Os-LBSO**, but not **Ru-LBSO**, demonstrated glutathione inhibition. These studies on **Ru-LBSO** and **Os-LBSO** complexes demonstrate the challenges of making progress toward the development for clinical use of organometallic complexes that contain multiple chiral centers. However, they offer exciting possibilities for discovery of novel drugs with new mechanisms of action.

## Graphical abstract

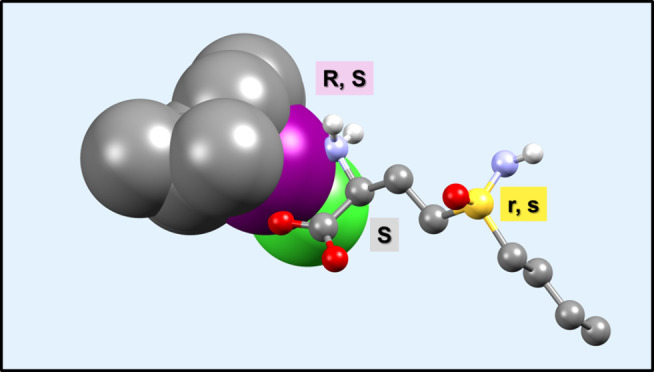

Half-sandwich organometallic complexes offer promise for the design of anticancer drugs with novel mechanisms of action to combat resistance with low side effects,^1–3^ including Ru­(II) and Os­(II) arene complexes.^4–8^ However, rational design requires knowledge of chemical reactivity of the complex during transport to the target site (e.g., hydrolysis and reactions with biomolecules) and in cells, including at its target sites. Moreover, the candidate metallodrug itself should be well characterized in terms of its chiral purity since different enantiomers can have different activities. For example, the R,R enantiomer of oxaliplatin is used clinically since it is more active than its S,S enantiomer.^9^

The intracellular tripeptide glutathione (γ-l-Glu-l-Cys-Gly, GSH) is highly abundant (ca. 2–7 mM) in cancer cells^10^ and most other cells. It plays an important role in maintaining the intracellular redox balance, as well as an antioxidant for reactive oxygen species (ROS) and binds strongly to many transition metal ions.^11^ The biosynthetic pathway for GSH involves the enzyme γ-glutamylcysteine synthetase (γ-GCS).^12^ *L*-BSO is a known inhibitor of γ-GCS and is already used in the clinic as a racemic mixture.^13^ It has a chiral carbon (S-configuration) but also a chiral sulfur (r/s). Therefore, we hypothesize that delivery of *L*-BSO by **Ru-LBSO** or **Os-LBSO** might reduce the levels of GSH in cells and reduce possible detoxification of Ru­(II)/Os­(II), which in turn could potentiate the effectiveness of reactive oxygen species (ROS) in destroying cells.

Here, we have synthesized dual-function organometallic anticancer complexes: [(*p*-cymene)­M­(XY)­Cl], XY = *L*-BSO, M = Ru^II^ (**Ru-LBSO**), Os^II^ (**Os-LBSO**), containing the amino acid *L*-buthionine sulfoximine (*L*-BSO), as well as their XY = glycine analogs (**Ru-Gly** and **Os-Gly**). These complexes might bind to DNA via reactive Ru/Os–Cl sites and also have potentially labile *N*,*O*-chelated amino acid ligands which might be released inside cells.^5,8^ The synthetic routes to the *L*-BSO complexes followed that reported previously for the glycine complexes (Scheme 1).^5,8^ 1D and 2D ^1^H and ^13^C NMR spectroscopy, high resolution mass spectrometry (HRMS), and HPLC were used to characterize Ru and Os glycine complexes. **Ru-Gly** was also characterized as a methanol solvate by single crystal X-ray diffraction (Figure S15).^8^

**Scheme 1:**
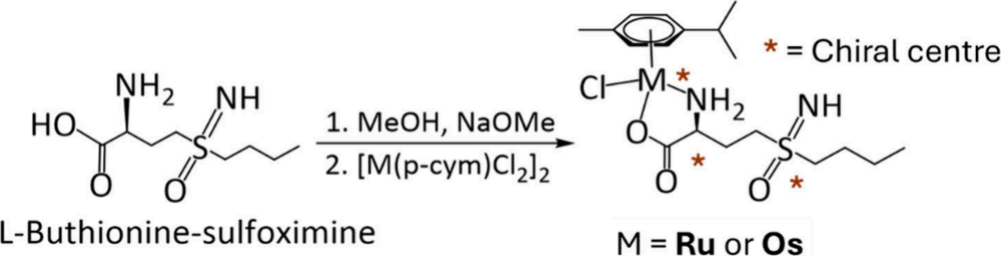
Synthetic Route for *p*-Cymene Ru­(II) and Os­(II) Chlorido Complexes^a^

a There are 3 chiral centers (*) in the product, one at metal center (R or S) and two on the ligand CH (S, fixed) and sulfur (s or r). Additionally, axial chirality arises from the orientation of the *p*-cymene relative to the N,O-chelated amino acid,^14^ but is not further considered here.

^1^H and ^13^C NMR spectra of the *L*-BSO complexes in methanol-*d*_4_ and D_2_O are complicated, even at 700 MHz for ^1^H (Figures S5 and S9), attributable to the presence of diastereomers (chiral centers at Ru/Os (R,S)) and *L*-BSO (*S* at carbon, *s* or *r* at sulfur). The ^1^H NMR spectra of **Os-LBSO** and **Ru-LBSO** in methanol-*d*_4_ are shown in Figures S5 and S9, respectively. The spectra of the two compounds are similar. Reasonable assignments for some of the peaks were possible based on ^1^H–^13^C HMBC cross-correlations (Figures S7 and S11) and 2D ^1^H–^1^H NOESY spectra (Figures S8 and S12). Assignments were also aided by the ^1^H NMR spectrum of *L*-BSO itself.^15^ The ^1^H spectra appear to be dominated by a set of peaks assignable to one major diastereomer. These are labeled a, b, etc in Figures S5 and S9. A minor set of peaks labeled a′, b′, etc. is assignable to a second diastereomer. Further very weak peaks may be assignable to two other less favored diastereomers.

The aqueous stability of **Os-LBSO** and **Ru-LBSO** complexes was studied (Figures S18–S21). Hydrolysis may be an important step in mechanism of action of chlorido half-sandwich Ru­(II) and Os­(II) complexes.^5,8^ Os­(II) is generally more kinetically inert than its lighter congener Ru­(II).^5,7,8^ The commonly slower ligand exchange rate of the Os­(II) complexes may result in a different mechanism of action as compared to their Ru­(II) analogues.

**Ru-LBSO** appeared to hydrolyze within 15 min when dissolved in a 3:7 v/v methanol-*d*_4_:D_2_O at 310 K as monitored by ^1^H NMR (Figure S19). Hydrolysis was confirmed with the reappearance of signals corresponding to the chlorido species due to partial reversal of hydrolysis after the addition of excess (130 mM) NaCl (Figure S21).

Hydrolysis of **Os-LBSO** was also studied under similar conditions. Two sets of peaks in a 70:30 ratio were observed in the aromatic region assignable to *p*-cymene protons 10 min after dissolution a 3:7 v/v methanol-*d*_4_:D_2_O at 310 K (Figure S18). After 36 h, the intensity ratio of these peaks changed to 60:40 (Figure S16). Addition of excess NaCl (130 mM) to the 36 h solution had little effect on the spectrum even after 24 h (Figure S20), suggesting that this complex does not readily hydrolyze and two sets of peaks are due to two prominent diastereomers. The interconversion between two diastereomers might arise from chelate ring opening and closing.

DFT calculations were performed to model **Ru-LBSO** and **Os-LBSO** taking into account the absolute configurations of the metal (R or S), the amino acid carbon (S), and the sulfur of *L*-BSO (r or s) giving four possible diastereomers for each of the two complexes. The results show the small energetic stabilization of the [S_M_] compared to the [R_M_] diastereomer (Figure 1) leading to an estimated *K*_eq_ which correlates with an experimental NMR value of ca. 2.3 (Figure S5). The absolute configuration of the S-atom appears to have little influence on the overall free energies of the diastereomers. A possible reason for the free energy difference between the R_M_ and S_M_ diastereomers is the interaction between an amine hydrogen and the chlorido ligand. The N–H···Cl distance is 2.96 Å for the energetically preferred [S_M_] diastereomer (Figure 1(c)) and 2.475 Å for the [R_M_] diastereomer (Figure 1(d)). This ca. 0.5 Å difference does not result in significant changes to the metal–ligand bond-lengths for the Os···Cl, Os···N, and Os···O bonds, yet it leads to somewhat longer M···*p*-cymene (centroid to metal distance) and *p*-cymene···Cl distances for the diastereomer of lower energy (Figure 1(c,d)).

**Figure 1:**
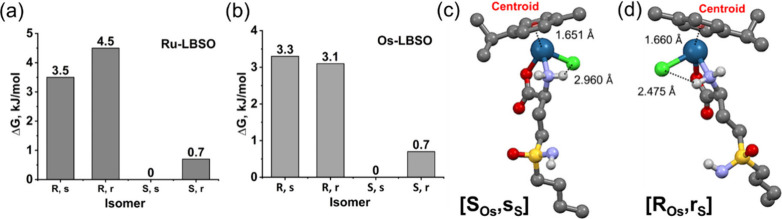
Difference in free energies (ΔG) at 298 K for four diastereoisomers of the (a) **Os-LBSO** and (b) **Ru-LBSO** complexes calculated by DFT with methanol modeled as the solvent. [R_M_,r_S_], [R_M_,s_S_], [S_M_,r_S_] and [S_M_,s_S_] are defined as [chirality at M­(II) (R_M_/S_M_), chirality at sulfur of *L*-BSO (r_S_/s_S_)]. (c,d) DFT optimized structures of diastereomers of **Os-LBSO**. (c)­[ R_Os_,r_S_] and (d)­[S_Os_,s_S_] diastereoisomers of Os-LBSO. Selected calculated interatomic distances: Os···Cl (2.435 Å), Os···N (2.163 Å), Os···O (2.089 Å), Os···centroid (1.660 Å) and centroid···Cl (3.408 Å) for [R_Os_,r_S_] (c); Os···Cl (2.438 Å), Os···N (2.165 Å), Os···O (2.092 Å), Os···centroid (1.651 Å) and centroid···Cl (3.634 Å) for­[S_Os_,s_S_] (d). The structures of the diastereomers of Ru-LBSO are similar (Figure S22).

The antiproliferative activity against human A549 lung, IGROV-1 ovarian, and HeLa cervical cancer cell lines and MRC5 normal lung fibroblasts was determined by the sulforhodamine B (SRB) assay.^16^ Table 1 shows that there is a remarkable and unusual pattern of activity. First, the Ru and Os glycine complexes are relatively non-toxic (IC_50_ > 200 μM) toward cancer cell lines and normal cells, consistent with published data.^5,8^ Second, only **Os-LBSO** and not **Ru-LBSO** is active against the IGROV-1 ovarian cancer cell line, to which it is reasonably potent with an IC_50_ value (14.6 μM) similar to that of cisplatin in the same cell line. IGROV-1 is known to be cisplatin-sensitive and subject to hypermutation.^17^ Hydrolysis experiments demonstrated that **Os-LBSO** is the most stable of these complexes and does not readily hydrolyze, suggesting that **Os-LBSO** is able to deliver *L*-BSO to intracellular targets (see Figures S18 and S19 for hydrolysis results). **Os-LBSO** is inactive toward lung cancer cells but is highly active toward normal lung cells, suggesting that factors other than *L*-BSO release may also be involved in the mechanism of action. Notably, **Os-LBSO** is also inactive toward HeLa cervical cancer cells.

**Table 1:** Antiproliferative Activity: IC_50_ Values for **Ru-LBSO**, **Os-LBSO**, **Ru-Gly**, **Os-Gly** and the Drug Cisplatin (CDDP) towards Human Cancer Cell Lines and Normal Lung Fibroblasts

		IC_50_/μM^a^				
Cell Line		Ru-LBSO	Ru-Gly	Os-LBSO	Os-Gly	CDDP
A549	Lung carcinoma	>200	>200	>200	>200	3.0 ± 0.2
MRC-5	Lung fibroblasts	4.3 ± 0.7	112 ± 4	11.8 ± 0.6	107 ± 2	13.5 ± 0.9
IGROV-1	Ovarian carcinoma	108 ± 8	>200	14.6 ± 0.5	>200	20.0 ± 4.8
HeLa	Cervical adenocarcinoma	>200	>200	>200	>200	

a IC_50_ determined by the SRB assay after 24 h treatment with complexes and 72 h recovery after removal of the complexes

We investigated further the activity of **Os-LBSO and Ru-LBSO** in the IGROV-1 ovarian cancer cell line by determining the percentage of IGROV-1 in cell cycle stages (G1, S, G2/M) after 24 h of treatment. **Os-LBSO** (100 μM) and **Ru-LBSO** (100 μM) significantly increased the level of G1 arrest (Figure 2). Intriguingly, both **Ru-Gly** and **Os-Gly** which were inactive in cancer cells notably showed little progression to G2/M; i.e., cell growth is blocked in G1/S phase and cells do not progress to mitosis. This observation is also worthy of further investigation since the low toxicity of the glycine complexes might make them suitable for use as e.g. antimetastatic agents. IGROV cells are highly migratory and express vitronectin and avβ3 integrin.^18^

**Figure 2:**
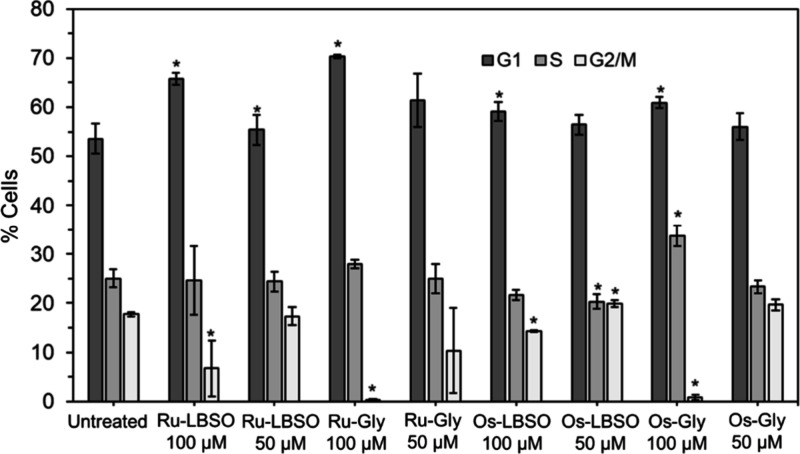
Effect of **Os**/**Ru-LBSO** and glycine complexes at 50 or 100 μM on the phases of the cell cycle of IGROV-1 human ovarian cancer cells compared with untreated (control) cells. Percentage of cells in G1 (black bar, cell growth), S (gray, DNA synthesis), G2/M, (white, growth/mitosis) as determined by flow cytometry after 24 h treatment with complexes. It is notable that the active complex **Os-LBSO** (100 μM) significantly increased G1 arrest. Standard deviations calculated for *N* = 3 *p-value <0.05 comparing to untreated sample. Representative flow cytometry plots are shown in Figure S23.

Apoptosis assays using annexin V and PI as markers were carried out to investigate possible modes of cell death. For the clinical drug cisplatin, apoptosis is the major mode of cell death.^19^ However, no significant differences were observed for treated cells (including **Os-LBSO**) compared to untreated cells after 24 h (Figure S24). Other mechanisms of cell death such as ferroptosis could be investigated in future work.^20^

A glutathione colorimetric detection assay (see section S2.13 in the Supporting Information) used to determine the level of intracellular GSH in IGROV-1 cells suggested that **Os-LBSO** lowers the level by ca. 50%; however, further measurements would be needed to confirm the statistical significance (Figure S25). This lowering was not observed for **Ru-LBSO** which is not active in this cell line. Other papers have shown a similar result by including *L*-BSO as an additional agent, therefore showing *L*-BSO remains inhibitory.^21^ This may be due to the stability of the **Os-LBSO** complex over the **Ru-LBSO** (discussed above), allowing for intact delivery.

Comet assays were carried out to determine whether the complexes caused DNA damage in single cells. Cisplatin and **Os-Gly** at 100 μM showed statistically significantly shorter comet tails than those for untreated cells. Cisplatin DNA-adducts have been shown previously to inhibit DNA migration through electrophoresis presenting as less tail movement.^22^ **Os-LBSO** has a trend toward longer comet tails but this was not significant (Figure S26). Although DNA damage may play a role in the **Os-LBSO** mechanism of action, it is likely to be multitargeting.

In summary, we have synthesized novel half-sandwich arene Ru­(II) and Os­(II) complexes containing the clinical drug *L*-BSO, an inhibitor of glutathione synthesis. The presence of chiral metal and *L*-BSO gives rise to a complicated mixture of species as detected by NMR and analyzed as four diastereomers on the basis of DFT calculations. These indicated a small preference for the S_M_ metal configuration with little influence from the chirality of the sulfur in *L*-BSO. Cytotoxicity screening toward human lung, ovarian, and cervical cancer cells and normal cells showed differences both between the **Ru-LBSO** and **Os-LBSO** complexes and the **Ru-Gly** and **Os-Gly** analogues. Interestingly, only **Os-LBSO** was active against IGROV-1 ovarian and HeLa cervical cancer cells. Both the **Ru-LBSO** and **Os-LBSO** complexes showed some activity in MRC-5 healthy fibroblasts.

Elucidation of the mechanisms of action of organometallic complexes is challenging especially when multiple chiral centers are present. It will be important in future work to investigate possible differences in lipophilicity and cellular uptake of these complexes, as well as to separate their diastereomers, and determine their chemical and biological activity, including stability toward epimerization, to understand further the intriguing selectivity of **Os-LBSO** toward specific cancer and normal cell lines.

## Supplementary Material


