# Supplementary material for: Multichiral Half-Sandwich Ru(II) and Os(II) Anticancer Complexes Containing a Glutathione Synthesis Inhibitor
Source: Organometallics. 2025 Dec 11;44(24):2829–33. doi: 10.1021/acs.organomet.5c00375 (PMC12728982; doi:10.1021/acs.organomet.5c00375)
Supplement: Supplementary file 1 [file om5c00375_si_001.pdf]

Supporting Information

Multi-chiral half-sandwich Ru(II) and Os(II) anticancer complexes containing a glutathione synthesis inhibitor

Pragya Kumari,[a] Hannah E. Bridgewater,[b,c] Sara Anisi,[b] Craig M. Whitehouse,[a] Adam J. Millett,[a] Prinessa Chellan,[d] Isolda Romero-Canelón,[e] Guy J. Clarkson,[a] Volker Schünemann,[f] Juliusz A. Wolny,[f] and Peter J. Sadler[a*]

[a]*Department of Chemistry, University of Warwick, Gibbet Hill Road, Coventry, CV4 7AL, UK*

[b]*Centre for Health and Life Sciences, Coventry University, Priory Street, CV1 5FB, UK*

[c]*School of Life Sciences, University of Warwick, Gibbet Hill Road, Coventry, CV4 7AL, UK*

[d] *Department of Chemistry and Polymer Science, Stellenbosch University, 7600 Matieland, Western Cape, South Africa*

[e] *School of Pharmacy, University of Birmingham, Birmingham B15 2TT, UK*

[f] *Department of Physics, RPTU University Kaiserslautern-Landau, Erwin-Schrödinger-Straße 46, 67663 Kaiserslautern, Germany*

Email: p.j.sadler@warwick.ac.uk

Table of Contents

1 Materials 3
2 Methods 3
2.1 Synthesis and characterisation 3
2.1.1 Synthesis of Ru-LBSO, [(*p*-cymene)Ru(*L*-BSO)Cl] 3
2.1.2 Synthesis of Os-LBSO, [(*p-cymene*)Os(*L*-BSO)Cl] 4
2.1.3 Synthesis of Ru-Gly, [(*p-cymene*)Ru(*Gly*)Cl] 5
2.1.4 Synthesis of Os-Gly, [(*p-cymene*)Os(*Gly*)Cl] 5
2.2 High Resolution Electrospray Ionization Mass Spectrometry (HR-ESI-MS) 6
2.3 NMR Spectroscopy 6
2.4 X-ray crystallography 6
2.5 Aqueous stability 7
2.6 High Performance Liquid Chromatography (HPLC) 7
2.7 Liquid chromatography-mass spectrometry (LC-MS) 7
2.8 Mammalian cell culture 8
2.9 Sulforhodamine (SRB) cytotoxicity assay 8
2.10 Cell Cycle Arrest 8
2.11 Apoptosis 9
2.12 Comet assay 9
2.13 Intracellular GSH 10

2.14 DFT Studies........10
3 References........28

Figures

Figure S1. 700 MHz ^{1}H NMR spectrum of Ru-Gly in methanol-d_4........13
Figure S2. 126 MHz 2D ^{13}C-APT NMR spectrum of Ru-Gly in methanol-d_4........13
Figure S3. 700 MHz ^{1}H NMR spectrum of Os-Gly in methanol-d_4........14
Figure S4. 126 MHz 2D ^{13}C-APT NMR spectrum of Os-Gly in DMSO-d_6........14
Figure S5. 700 MHz ^{1}H NMR spectrum of Os-LBSO in methanol-d_4.........15
Figure S6. 125 MHz 2D ^{13}C-APT NMR spectrum of Os-LBSO in methanol-d_4.........15
Figure S7. 2D ^{13}C (125 MHz) -^{1}H HMBC spectrum of Os-LBSO in methanol-d_4........16
Figure S8. 500 MHz 2D ^{1}H-^{1}H NOESY NMR spectrum of Os-LBSO in methanol-d_4.........16
Figure S9. 700 MHz ^{1}H NMR spectrum of Ru-LBSO in methanol-d_4........17
Figure S10. 125 MHz 2D ^{13}C-APT NMR spectrum of Ru-LBSO in methanol-d_4.........17
Figure S11. 2D ^{13}C (125 MHz) -^{1}H HMBC spectrum of Ru-LBSO in methanol-d_4........18
Figure S12. 500 MHz 2D ^{1}H-^{1}H NOESY NMR spectrum of Ru-LBSO in methanol-d_4.........18
Figure S13. ESI-MS spectrum of Os-LBSO in MeOH.........19
Figure S14. ESI-MS spectrum of Ru-LBSO in MeOH.........19
Figure S15. X-ray crystal structure of Ru-Gly·CH_3OH.........20
Figure S16. LC-MS of 100 µM Ru(II) L-BSO (acetonitrile : water , 0.1% formic acid).........21
Figure S17. HPLC of Os-LBSO........22
Figure S18. 400 MHz ^{1}H NMR spectra of Os-LBSO (2 mM) in 3:7 v/v methanol-d_4 : D_2O recorded 10 min and 36 h after dissolution at 310 K.........23
Figure S19. 400 MHz ^{1}H NMR spectra of Ru-LBSO (2 mM) in 3:7 v/v methanol-d_4 : D_2O recorded 10 min and 36 h after dissolution at 310 K.........23
Figure S20. 400 MHz ^{1}H NMR spectrum of Os-LBSO after addition of NaCl (130 mM) to 36 h sample in 3:7 v/v methanol-d_4 : D_2O........24
Figure S21. 400 MHz ^{1}H NMR of Ru-LBSO after addition of NaCl (130 mM) to 36 h sample in 3:7 v/v methanol-d_4 : D_2O.........24
Figure S22. DFT optimised structures of diastereomers of Ru-LBSO........25
Figure S23. Representative flow cytometry plots showing the effect of Os/Ru L-BSO and glycine complexes at 50 or 100 µM on the phases of the cell cycle of IGROV-1 human ovarian cancer cells........25
Figure S24. Effect of Os/Ru L-BSO and glycine complexes at 50 or 100 µM on the mode of death of IGROV-1........26
Figure S25. Effect of Ru/Os L-BSO at 50 µM complexes on the intracellular glutathione concentration in IGROV-1........26
Figure S26. % DNA in comet tail for IGROV-1 cells........27

1 Materials

α-Terpinene (≥ 85%), sodium methoxide (95%), ruthenium (III) chloride hydrate (≥ 99.98%), osmium (III) chloride hydrate (≥ 99.9%), trifluoroacetic acid (99%), anhydrous methanol (99.8%), deuterium oxide (99.9%), silver nitrate (≥ 99.9%), dichloromethane (≥ 99%) and reduced glutathione (≥ 98%) were purchased from Sigma-Aldrich. Sodium chloride, 1,4-dioxane (≥ 99.9%), acetonitrile (HPLC grade), water (HPLC grade) were purchased from Fischer Scientific, *L*-buthionine sulfoximine (≥ 97%) from Sigma-Aldrich, glycine (analytical grade) from BDH Chemicals. Methanol-d_4 (≥ 99.8%) and dimethyl sulfoxide-d_6 were purchased from Sigma-Aldrich. The dimers, dichloro(*p*-cymene)ruthenium(II) dimer and dichloro(*p*-cymene)osmium(II) dimer were synthesised using synthetic methods.[1]

Ru-Gly ([(*p-cymene*)Ru(*Gly*)Cl]) and Os-Gly ([(*p-cymene*)Os(*Gly*)Cl]) were synthesised using published procedures.[2,3]

2 Methods

2.1 Synthesis and characterisation

2.1.1 Synthesis of Ru-LBSO, [(*p*-cymene)Ru(*L*-BSO)Cl]

L-BSO (92.6 mg, 0.416 mmol, 2.5 mol eq) and sodium methoxide (22.5 mg, 0.416 mmol, 2.5 mol eq) in dry methanol (10 mL) were under N_2 gas for 1 h. [(*p*-cymene)$RuCl_2$]$_2$ (101.2 mg, 0.165 mmol, 1 mol eq) was then added and the reaction mixture was stirred under N_2 gas for 24 h. The solvent was removed via rotary evaporation, to leave an orange-yellow oil which was dissolved in dichloromethane (10 ml) causing NaCl to precipitate. The NaCl was removed by filtration and the solvent evaporated from the filtrate. The oily residue was redissolved in dichloromethane (1 ml) and hexane added (20 ml). A yellow precipitate formed was isolated

by centrifugation and drying overnight in a vacuum desiccator. ^{1}H NMR (700 MHz, methanol-*d$_4$*) δ 5.76 (d, 1H), 5.64 (d, 1H), 5.62 (d, 1H), 5.43 (d, 1H), 3.66 (t, 2H), 3.60 – 3.56 (m, 2H), 3.56 – 3.49 (m, 2H), 2.89 – 2.81 (m, 1H), 2.80 – 2.74 (m, 1H), 2.33 – 2.28 (m, 2H), 2.18 (s, 3H), 1.90 – 1.83 (m, 2H), 1.56 – 1.52 (m, 2H), 1.34 – 1.30 (m, 6H), 1.02 (m, 3H). ^{13}C NMR (126 MHz, methanol-*d$_4$*) δ 183.64, 102.96, 98.91, 84.56, 82.65, 82.53, 82.06, 56.52, 55.46, 52.02, 32.05, 26.47, 25.15, 22.77, 22.57, 22.44, 18.09, 13.81. $C_{18}H_{31}N_2O_3RuS$ $(M\text{-}Cl)^+$ 457.1086 m/z, found 457.1084 m/z. Yield: 58%. HPLC purity >95%.

2.1.2 Synthesis of Os-LBSO, [(*p-cymene*)Os(*L*-BSO)Cl]

L-BSO (72.2 mg, 0.327 mmol, 2.5 mol eq) and sodium methoxide (17.6 mg, 0.327 mmol, 2.5 mol eq) in dry methanol (10 mL) were under N_2 gas for 1 h. [(*p*-cymene)$OsCl_2$]$_2$ (104.2 mg, 0.131 mmol, 1 mol eq) was then added and the reaction mixture was stirred under N_2 gas for 24 h. The solvent was removed via rotary evaporation, to leave an orange-yellow oil which was dissolved in dichloromethane (10 ml) causing NaCl to precipitate. The NaCl was removed by filtration and the solvent evaporated from the filtrate. The oily residue was redissolved in dichloromethane (1 ml) and hexane added (20 ml). A yellow precipitate formed was isolated by centrifugation and drying overnight in a vacuum desiccator. ^{1}H NMR (700 MHz, methanol-*d$_4$*) δ 6.05 (d, 1H), 5.95 (d, 1H), 5.86 (d, 1H), 5.71 (d, 1H), 3.78 – 3.76 (m, 2H), 3.70 – 3.62 (m, 2H), 3.62 – 3.55 (m, 2H), 2.98 – 2.90 (m, 1H), 2.71 – 2.65 (m, 1H), 2.49 – 2.43 (m, 2H), 2.22 (s, 3H), 1.88 – 1.83 (m, 2H), 1.56 – 1.48 (m, 2H), 1.31 (dd, 6H), 1.02 (m, 3H). ^{13}C NMR (126 MHz, methanol-*d$_4$*) δ 186.59, 92.71, 89.89, 76.06, 73.32, 72.95, 71.95, 55.91, 55.63, 52.66, 32.54, 26.33, 25.12, 23.13, 23.10, 22.43, 18.30, 13.78. $C_{18}H_{31}N_2O_3OsS$ $(M\text{-}Cl)^+$ 547.1667 m/z, found 547.1663 m/z. Yield: 54%. HPLC purity >95%.

2.1.3 Synthesis of Ru-Gly, [(*p-cymene*)Ru(*Gly*)Cl]

$[RuCl_2(p\text{-cymene})]_2$ (M. wt. 612.4 g/mol) (0.100 g, 0.163 mmol), glycine (0.0244 g, 0.326 mmol), and potassium *tert*-butoxide (0.0365 g, 0.326 mmol) were dissolved in 5 ml of methanol and stirred for 30 min. The volume of solution was reduced to ~2 ml, and dichloromethane was added. Potassium chloride precipitate appeared and was filtered off. Diethyl ether was added to the filtrate and the desired product was precipitated. The latter was filtered and dried under vacuum to give an orange-yellow solid. ^{1}H NMR (700 MHz, methanol-*d4*) δ_H 6.25 (s, 1H), 5.66 (d, 1H), 5.63 (d, 1H), 5.46 (d, 1H), 5.42 (d, 1H), 4.12 (s, 1H), 3.12 – 3.02 (m, 2H), 2.87 – 2.78 (m, 1H), 2.18 (s, 3H), 1.31 (dd, 6H). ^{13}C NMR (176 MHz, methanol-*d4*) δ 184.29, 102.05, 97.57, 83.91, 81.75, 81.54, 80.73, 45.95, 32.13, 22.92, 22.47, 18.29. HRMS calcd for $C_{12}H_{18}NO_2Ru$ $(M\text{-}Cl)^+$ 310.0381 m/z, found 310.0379 m/z. Yield: 73%. Single crystals of [(*p-cymene*)Ru(*Gly*)Cl]·CH_3OH (**Ru-Gly·MeOH**) were grown from methanol at 277 K.

2.1.4 Synthesis of Os-Gly, [(*p-cymene*)Os(*Gly*)Cl]

$[OsCl_2(p\text{-cymene})]_2$ (M. wt. 790.7 g/mol) (0.128 g, 0.163 mmol), glycine (0.0244 g, 0.326 mmol), and potassium *tert*-butoxide (0.0365 g, 0.326 mmol) were dissolved in 5 ml of methanol and stirred for 30 min. The volume of solution was reduced to ~2 ml, and dichloromethane was added. Potassium chloride precipitate appeared and was filtered off. Diethyl ether was added to the filtrate and the desired product was precipitated. The latter was filtered and dried under vacuum to give a yellow solid. ^{1}H NMR (700 MHz, DMSO-*d6*) δ_H 6.91 (s, 1H), 5.95 (d, 1H), 5.88 (d, 1H), 5.72 (d, 1H), 5.65 (d, 1H), 4.80 (s, 1H), 2.97 – 2.88 (m, 1H), 2.84 – 2.75 (m, 1H), 2.65 – 2.58 (m, 1H), 2.10 (s, 3H), 1.20 (dd, 6H). ^{13}C NMR (176 MHz,

DMSO d_6) δ 182.64, 88.95, 86.00, 73.47, 71.15, 69.51, 68.66, 43.43, 30.80, 22.96, 22.37, 17.97. HRMS calcd for $C_{12}H_{18}NO_2Os$ $(M - Cl)^+$ 400.0949 m/z, found 400.0947 m/z. Yield: 75%.

2.2 High Resolution Electrospray Ionization Mass Spectrometry (HR-ESI-MS)

HR-ESI-MS analysis was carried with a Bruker MaXis plus Q-TOF mass spectrometer equipped with electrospray ionisation source. The mass spectrometer was operated in electrospray positive ion mode with a scan range 50 m/z-2,400 m/z. Calibration was carried out with sodium formate (10 mM) before the analysis.

2.3 NMR Spectroscopy

All 1D ^{1}H, 2D ^{13}C-APT, 2D ^{13}C-^{1}H and 2D ^{1}H-^{1}H NMR spectra were obtained at 298 K (unless stated otherwise) on Bruker Avance 500 MHz or Bruker Avance NEO 700 MHz spectrometers. 500 MHz 2D ^{1}H-^{1}H NOESY NMR spectra were acquired the standard Bruker pulse sequence noesygpph with gradient selection. The relaxation delay (D1) was 1.75 s, and the mixing time (D8) was 600 ms. 1D ^{1}H NMR and ^{13}C chemical shifts were internally referenced to the residual signals of the solvents. The data were processed using MestreNova NMR analysis software.

2.4 X-ray crystallography

A suitable crystal of [(*p-cymene*)Ru(*Gly*)Cl]·CH_3OH (**Ru-Gly·MeOH**) was selected and mounted on a glass fibre with Fomblin oil and placed on a Rigaku Oxford Diffraction SuperNova diffractometer with a dual source (Cu at zero) equipped with an AtlasS2 CCD area detector. The crystal was kept at 150± 2 K during data collection.[4] Using Olex2[5], the structure was solved with the SHELXT[4] structure solution program using Intrinsic Phasing and refined with the SHELXL[6] refinement package using Least Squares minimisation. X-ray crystallographic data for [(*p-cymene*)Ru(*Gly*)Cl]·CH_3OH (**Ru-Gly·MeOH**) has been deposited in the Cambridge Crystallographic Data Centre under the accession number CCDC 2479790. X-ray

crystallographic data in CIF format are available from the Cambridge Crystallographic Data Centre (http://www.ccdc.cam.ac.uk/). The data were processed using Mercury 4.0. software.[7]

2.5 Aqueous stability

Ru-LBSO and Os-LBSO (ca. 2 mM) were dissolved in 5% methanol-d_4 - 95% D_2O and their aqueous stability was studied using NMR spectroscopy for up to 36 h at 298 K. Chemical shifts were referenced to TMS via a 1,4-dioxane internal standard.

2.6 High Performance Liquid Chromatography (HPLC)

Solutions of metal complexes (100 µM) were prepared in 1:10 v/v acetonitrile: deionized water, filtered through Iso-DiscTM filters (PTFE-4-4 4 mm x 0.45 µm), and then injected (50 µL) into the HPLC instrument. % Purity was calculated from the peak area of the complex peak in the HPLC chromatogram. 254 nm was selected as the detection wavelength with reference wavelengths set to 360 nm and 510 nm, and the stationary phase was an Agilent ZORBAX Eclipse Plus C18 250 x 4.6 mm (pore size of 5 µm). The mobile phase consisted of solvent A as HPLC grade H_2O + 0.1% TFA, and solvent B as HPLC grade MeCN + 0.1% TFA. The following solvent gradient was used with a flow rate of 1 mL/min. The data were analysed using ChemStation software. See Table S3 for HPLC gradient contitions.

2.7 Liquid chromatography-mass spectrometry (LC-MS)

LC-MS experiments were carried out using a Bruker Amazon X+ instrument coupled with an Agilent Technologies 1200 series HPLC instrument. The same HPLC column, method and conditions were used as shown in Section 2.6 with the exception that formic acid was used as the additive for LC-MS instead of TFA. Samples (20 µL) were injected and the mass spectrometer was operated in electrospray positive mode with a scan range of 50-2000 m/z.

2.8 Mammalian cell culture

The human ovarian cancer cell line, IGROV-1; the human cervical cancer cell line, HeLa; the human adenosarcoma cell line, A450; and the human non-cancerous lung fibroblast cell line, MRC5 were cultured in DMEM supplemented with 10% FBS and 100 units/mL of penicillin and 100 µg/mL of streptomycin (1x pen/strep). All cell lines were incubated at 310 K with 5% CO_2 and 95% humidity for routine maintenance and during drug treatment.

2.9 Sulforhodamine (SRB) cytotoxicity assay

Cells were seeded at 5 × 10^3 cells per well and incubated at 310 K for 48 h. Stock solutions (200 µM) of the complexes were prepared by dissolving the compound in 5% (v/v) DMSO followed by dilution with DMEM and serial dilutions with DMEM were prepared (0.1 µM-200 µM). The cells were exposed to the drug for 24 h. The drug was then removed, and cells were allowed to recover for 72 h, in drug-free media. Cell viability was then determined by the SRB colorimetric assay. The solubilised dye absorbance was analysed on a microplate reader (Thermo Scientific Multiskan FC with Scanit software 4.1) at 470 nm. Data were normalized using Microsoft Excel, and dose-response curves fitted using Origin 2016. The IC_{50} values determined by this assay refer to 50% inhibition of cell viability as 50% protein content.[8]

2.10 Cell Cycle Arrest

IGROV-1 cells (1 x 10^6 cells per well) were seeded in a 6-well plate and incubated for 24 h at 310 K. Stock solutions (200 µM) of the complexes were prepared by dissolving the compound in 5% (v/v) DMSO followed by dilution with DMEM and then diluted to desired concentrations. All complexes (50 µM and 100 µM) were added to the wells and incubated for 24 h. Cell pellets were collected using 0.25% trypsin and centrifuged (500 x g, 5 mins at 4oC). Then washed in PBS and fixed with 100% ice-cold ethanol for 20 min on ice. The ethanol

was aspirated, cells washed in ice-cold PBS and then stained with 0.2 mg/mL RNase, 0.05 mg/mL propidium iodide (PI) in PBS for 25 min in the dark and on ice. Excess dye was removed, and cells were re-suspended in PBS. Samples were analysed by flow cytometry using the BD FACSMelody™ Cell Sorter (R653880001) and data analysis on FlowJo™ v10 Software (BD Life Sciences).[9]

2.11 Apoptosis

IGROV-1 cells (1 x 10^6) were seeded in a 6-well plate and incubated for 24 h at 310 K. Stock solutions (200 µM) of the complexes were prepared by dissolving the compound in 5% (v/v) DMSO followed by dilution with DMEM and then diluted to desired concentrations. All complexes (50 µM and 100 µM) were added to the wells and incubated for 24 h. Cell pellets were collected using 0.25% trypsin and centrifuged (500 x g, 5 mins at 277 K). Cell pellets were washed with PBS and then stained with the commercial Apoptosis Detection Kit–FITC Annexin V and PI (BioLegend; San Diego, USA), as per manufacturer's instructions. Briefly, a staining solution of FITC Annexin V (1 unit/100 µL) diluted 1 in 100 and 1 µg/mL PI was added to the cells (30 min, room temperature). Excess dye was removed, and cells were re-suspended in PBS. Samples were analysed by flow cytometry using the BD FACSMelody™ Cell Sorter (R653880001) and data analysis on FlowJo™ v10 Software (BD Life Sciences).[9]

2.12 Comet assay

IGROV-1 cells (8×10^5 cells per well) were seeded into 6-well plates and incubated for 24 h. Test compounds (50 µM and 100 µM) were added to the wells and incubated at 310 K for 24 h. Cells were enzymatically detached using trypsin and cell concentration adjusted to 2×10^4 cells /mL in ice-cold PBS. 0.4 mL of cell suspension was added to 1.2 mL of 1% low melting point agarose in H_2O, then immediately added to microscope slides pre-coated in 1% agarose.

Once the agarose had set, the slides were submerged in alkaline lysis solution (1.3 M NaCl, 100 mM Na_2EDTA, 0.1% sodium lauryl sarcosinate, 0.26 M NaOH pH > 13) and incubated overnight at 277 K in the dark. The following day the slides were rinsed for 20 min three times in the electrophoresis solution (0.03 mM NaOH, 2mM Na_2EDTA pH 12.3). The slides were then placed into the electrophoresis tank with fresh electrophoresis buffer, 1-2 mm above the slides. Electrophoresis was run at 18.3 V for 25 min. Once electrophoresis was complete, slides were rinsed in distilled water and placed in 2.5 µg/mL PI for 20 min. After staining, cells were rinsed in distilled water to remove excess stain. Twenty comet images per sample were taken by confocal microscopy and analysed by OpenComet.[10]

2.13 Intracellular GSH

1 x 10^6 IGROV-1 cells were seeded in a 6-well plate and incubated for 24 h at 310 K. All complexes (50 µM and 100 µM) were added to the wells and incubated for 24 h. After a 1x PBS wash, the commercial Glutathione Colorimetric Detection Kit (EIAGSHC/Invitrogen; Massachusetts, USA) was carried out, as per manufacturer's instructions.

2.14 DFT Studies

DFT calculations were performed to model Ru-LBSO and Os-LBSO. The absolute configuration of the metal centre was either R or S. The chirality of the amino acid carbon was S, while the chirality of the sulfur atom was r or s, giving four possible diastereomers. The calculations were performed with the TPSS functional[11] and the tzvp basis set[12] with the D3 dispersion correction of Grimme[13]. Methanol was modelled as solvent using the IEFPCM approach[14]. Gaussian 16 packet was used for modelling[15]. The pdb files of the optimised structures are available as supporting information with the following file names mentioned in Table S1.

Tables

Table S1. DFT optimised structures, as cif files for diastereomers of Os-LBSO and Ru-LBSO. $[R_M,r_S]$,$[R_M,s_S]$ $[S_M,r_S]$ and $[S_M,s_S]$ are defined as [chirality at M(II) (R_M/ S_M), chirality at sulphur of L-BSO (r_S /s_S)]. The results of the calculations are shown in Figure 3 in the main paper.

Diastereomer	***File name***
$[R_{Os}, r_S]$	opt_Os-LBSO_R-Os-r-S
$[R_{Os}, s_S]$	opt_Os-LBSO_R-Os-s_S
$[S_{Os}, r_S]$	opt_Os-LBSO_S-Os-r-S
$[S_{Os}, s_S]$	opt_Os-LBSO_S-Os-s-S
$[R_{Ru}, r_S]$	opt_Ru-LBSO_R-Ru-r-S
$[R_{Ru}, s_S]$	opt_Ru-LBSO_R-Ru-s_S
$[S_{Ru}, r_S]$	opt_Ru-LBSO_S-Ru-r-S
$[S_{Ru}, s_S]$	opt_Ru-LBSO_S-Ru-s-S

Table S2. Crystallographic data for **Ru-Gly·MeOH**. The asymmetric unit contains two crystallographically independent but chemically identical complexes and two molecules of methanol (Figure S15). There are four of these asymmetric units in the unit cell.

	**Ru-Gly·MeOH**
Formula	$C_{12}H_{22}ClNO_3Ru$
Molar mass /g mol^{-1}	376.83
Density /mg mm^{-3}	1.593
Crystal system	monoclinic
Crystal dimensions /mm	0.18 × 0.03 × 0.005 yellow block
Space group	$P2_1/c$
a/Å	12.94429
b/Å	10.09868
c/Å	24.10751
α/deg	90
β/deg	94.3248
γ/deg	90
T/K	150
Z	8
R [$F > 4\sigma(F)$]	0.0217
R_w	0.0546
GOF	1.169
Δρ max and min /eÅ^{-3}	0.45/-0.62

Table S3. HPLC gradient conditions.

**Time (min)**	**%(B)**
0	10
30	90
35	90
36	10
50	10

Figures

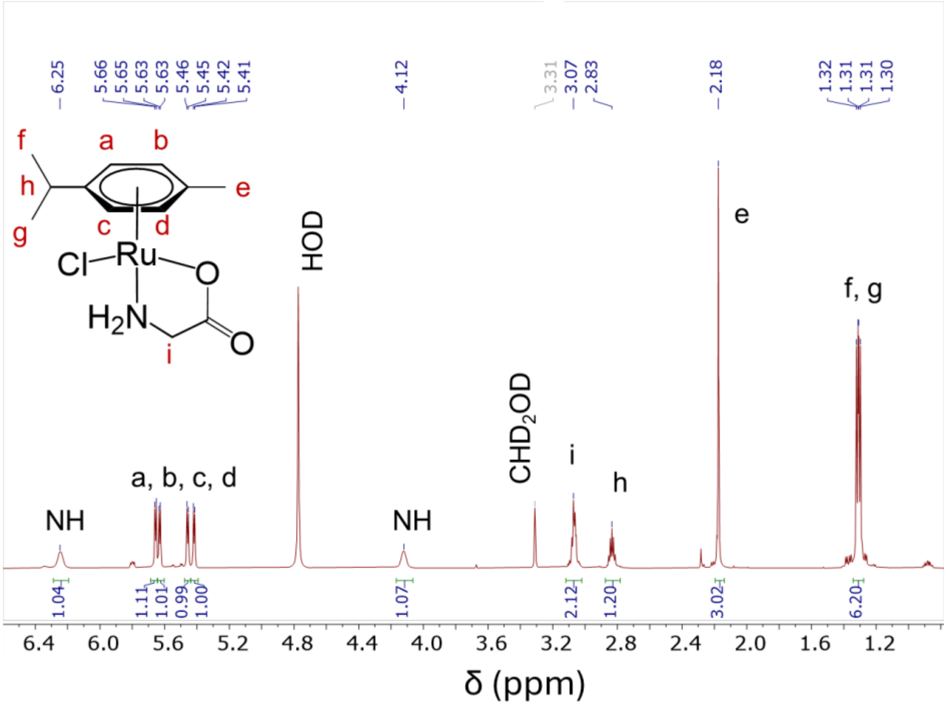

Figure S1. 700 MHz ^{1}H NMR spectrum of Ru-Gly in methanol-d_4 with the assignments shown.

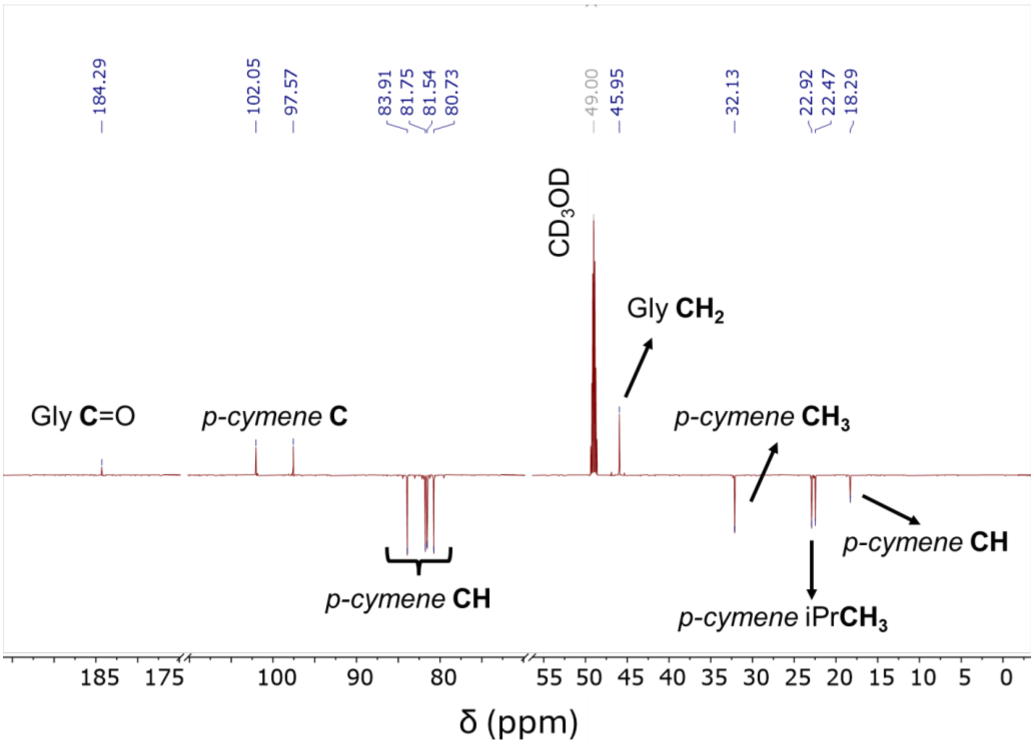

Figure S2. 126 MHz 2D ^{13}C-APT NMR spectrum of Ru-Gly in methanol-d_4 (CD_3OD). C and CH_2 peaks upright, CH and CH_3 peaks inverted.

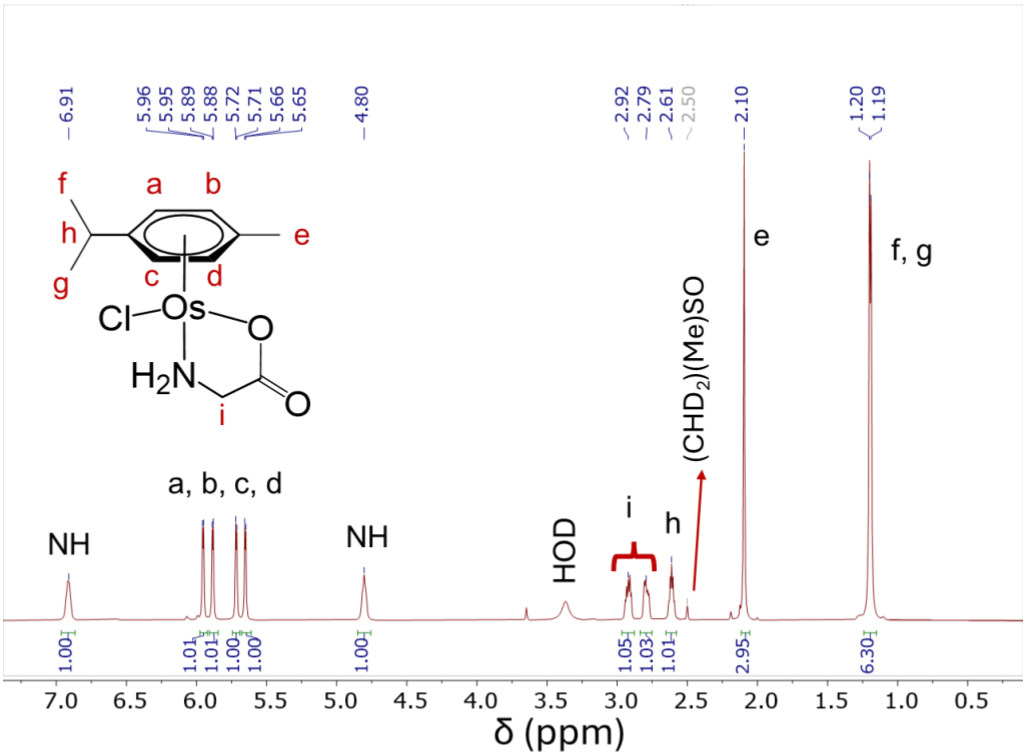

Figure S3. 700 MHz ^{1}H NMR spectrum of Os-Gly in methanol-d_4 with the assignments shown.

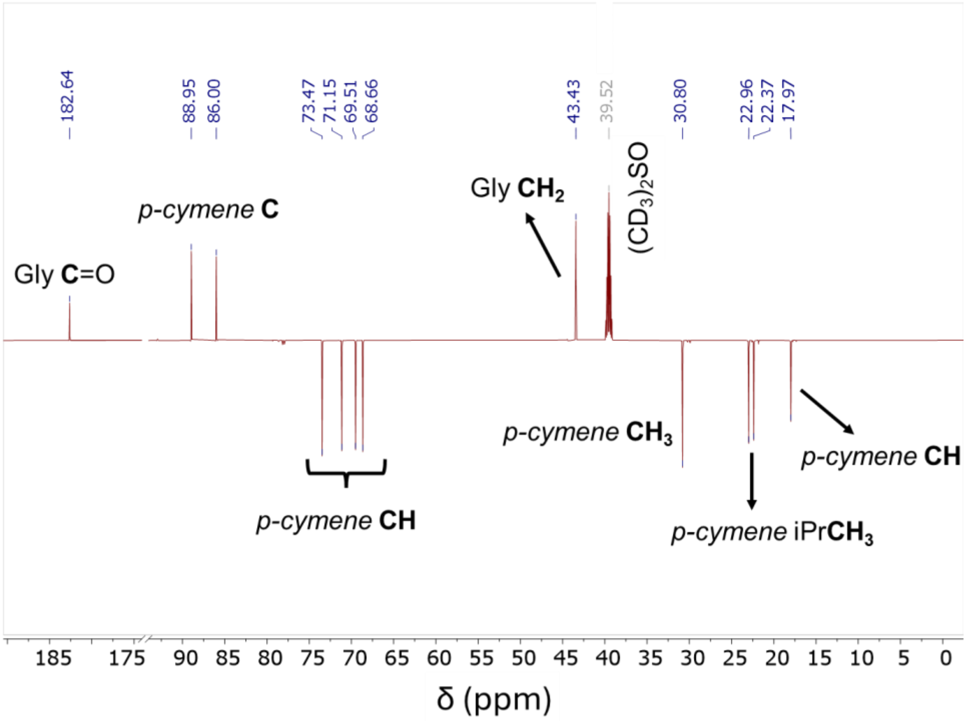

Figure S4. 126 MHz 2D ^{13}C-APT NMR spectrum of Os-Gly in DMSO-d_6 ($(CD_3)_2SO$). C and CH_2 peaks upright, CH and CH_3 peaks inverted.

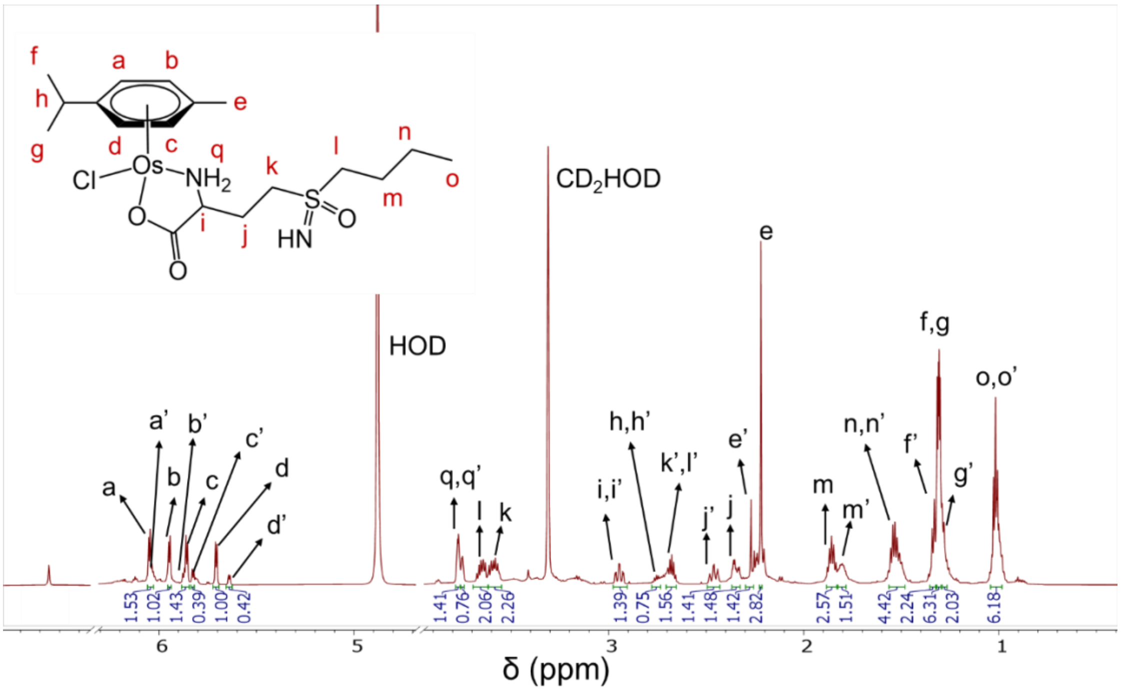

Figure S5. 700 MHz ^{1}H NMR spectrum of **Os-LBSO** in methanol-d$_4$, together with ^{1}H labelling, undashed a,b, etc major set of peaks and dashed a',b', etc minor set of peaks assignable to two diastereomers (Figure 1). The ratio of these diastereomers was estimated to be ca. 70:30 by peak integration.

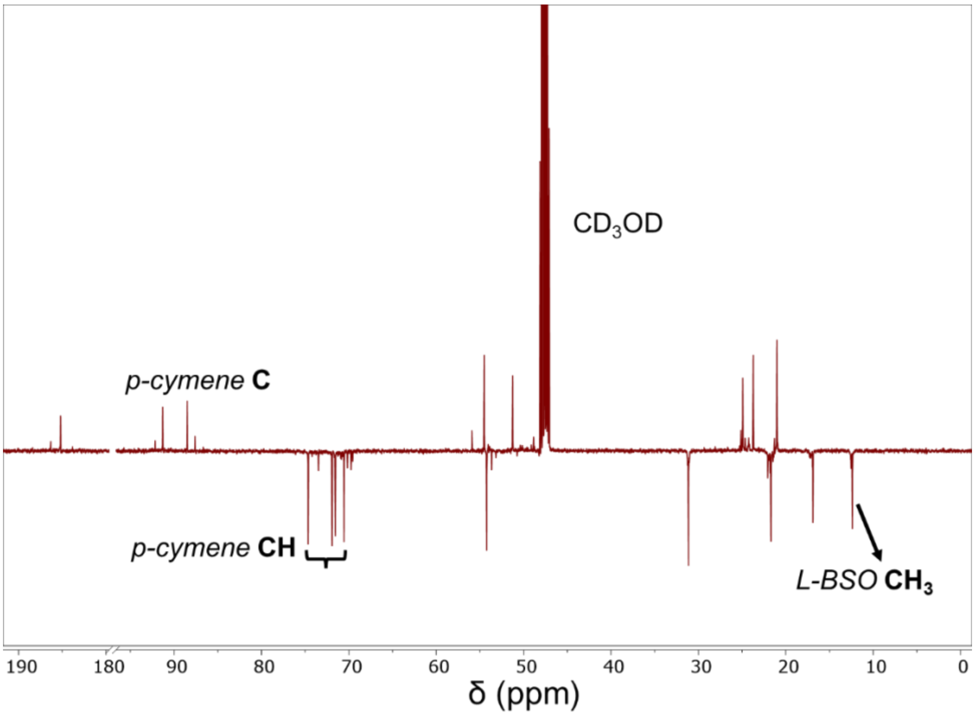

Figure S6. 125 MHz 2D ^{13}C-APT NMR spectrum of Os-LBSO in methanol-d$_4$ (CD_3OD). C and CH_2 peaks upright, CH and CH_3 peaks inverted. The presence of two diastereomers (as in the proton NMR spectrum) is apparent.

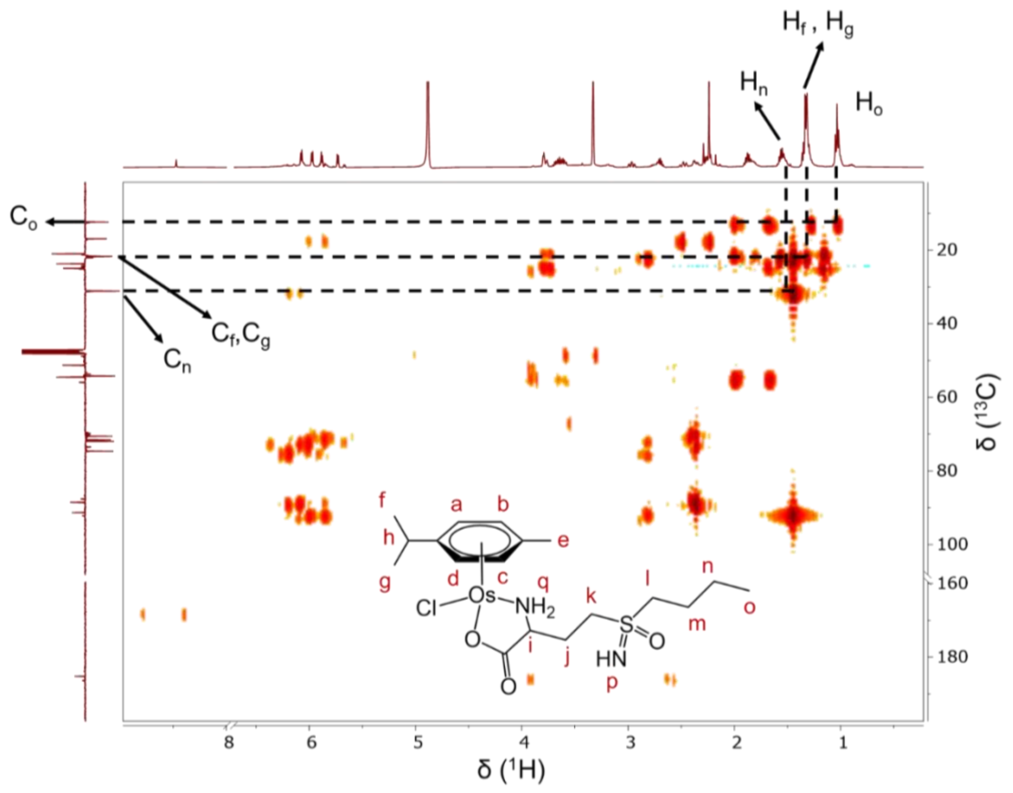

Figure S7. 2D ^{13}C (125 MHz) -^{1}H HMBC spectrum of Os-LBSO in methanol-d4 showing $H_f - C_f$, $H_g - C_g$, $H_o - C_o$ and $H_n - C_n$ cross peak assignments.

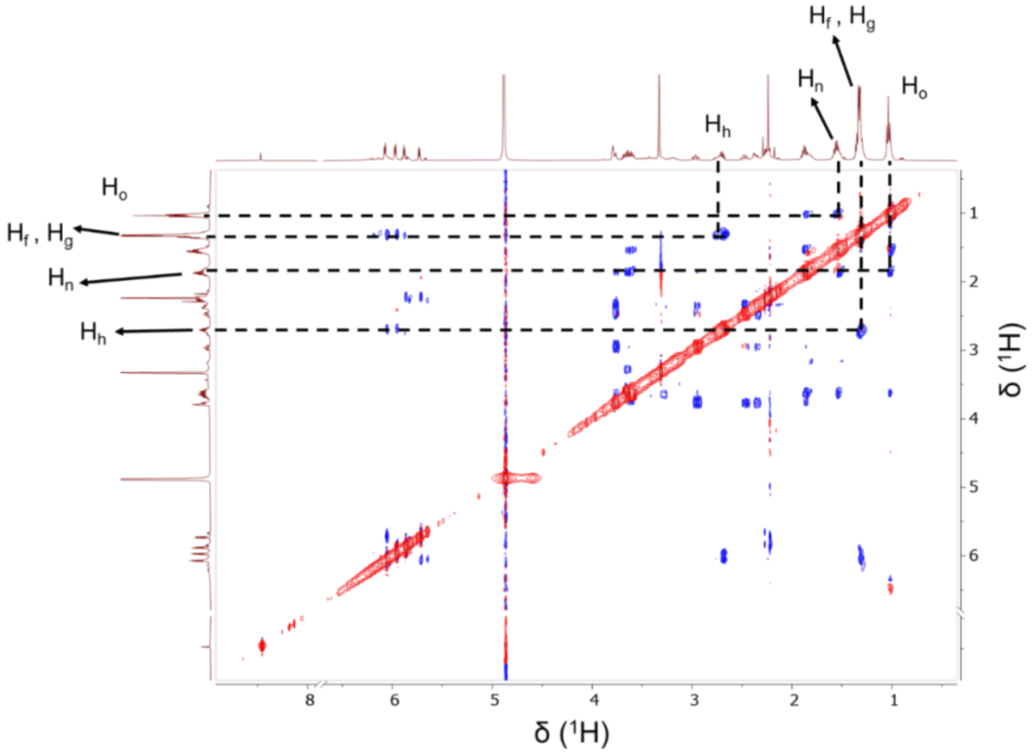

Figure S8. 500 MHz 2D ^{1}H-^{1}H NOESY NMR spectrum of Os-LBSO recorded at 0.6 sec mixing time in methanol-d4 showing H_n-H_o , H_h-H_f and H_h-H_g cross peak assignments.

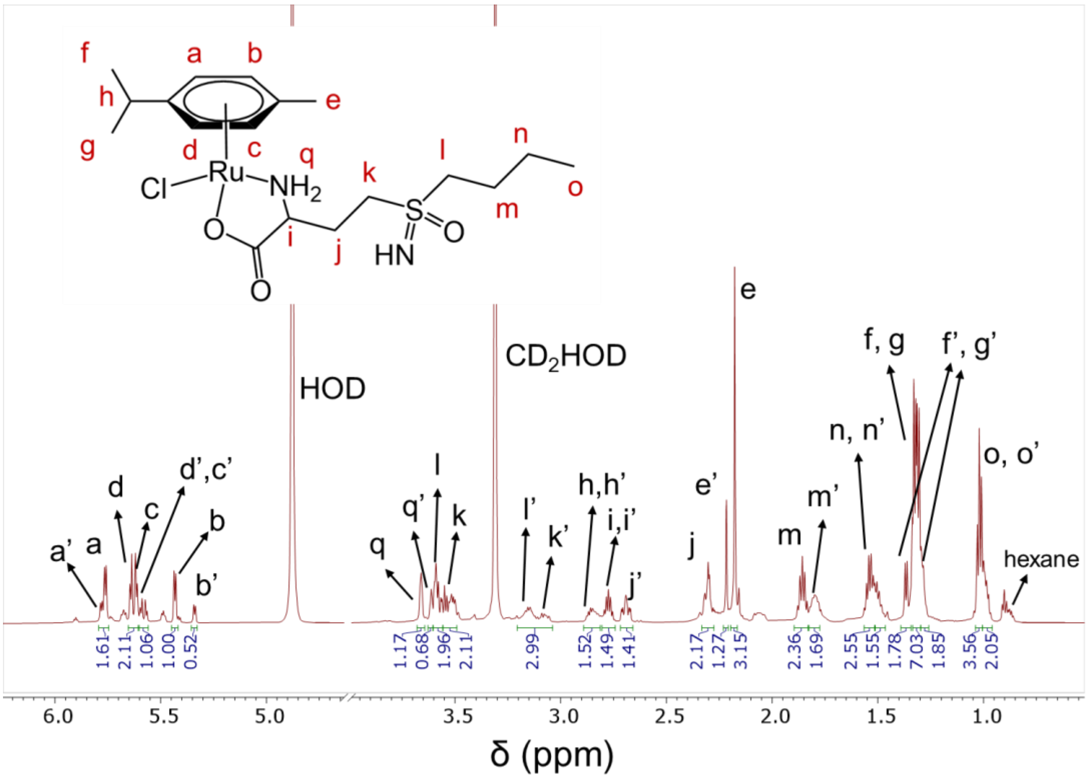

Figure S9. 700 MHz ^{1}H NMR spectrum of Ru-LBSO in methanol-d4 with the assignments shown. As for Os-LBSO (Figure 1), the spectrum contains peaks for a mixture of two diastereomers (undashed and dashed). Due to the chirality, all protons are magnetically non-equivalent. Integration shows the ratio of diastereomers is 70:30, as for Os-LBSO.

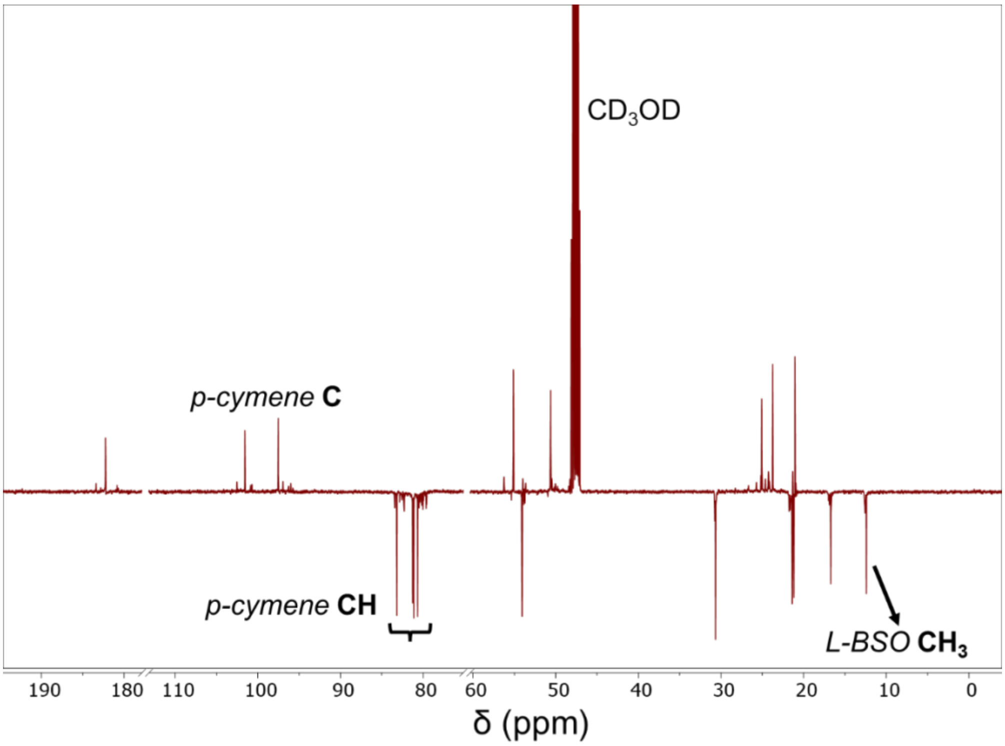

Figure S10. 125 MHz 2D ^{13}C-APT NMR spectrum of Ru-LBSO in methanol-d4. C and CH_2 peaks upright, CH and CH_3 peaks inverted. The presence of two diastereomers (as in the proton NMR spectrum) is apparent as for Os-LBSO.

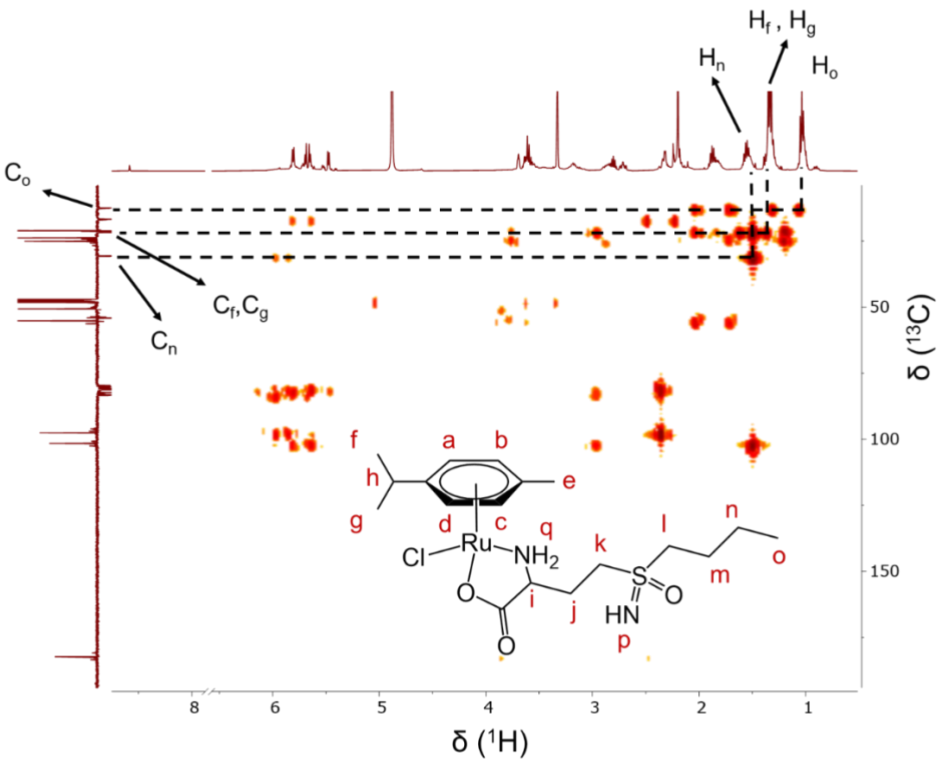

Figure S11. 2D ^{13}C (125 MHz) -^{1}H HMBC spectrum of Ru-LBSO in methanol-d$_4$ showing $H_f – C_f$, $H_g – C_g$, $H_o – C_o$ and $H_n – C_n$ cross peak assignments.

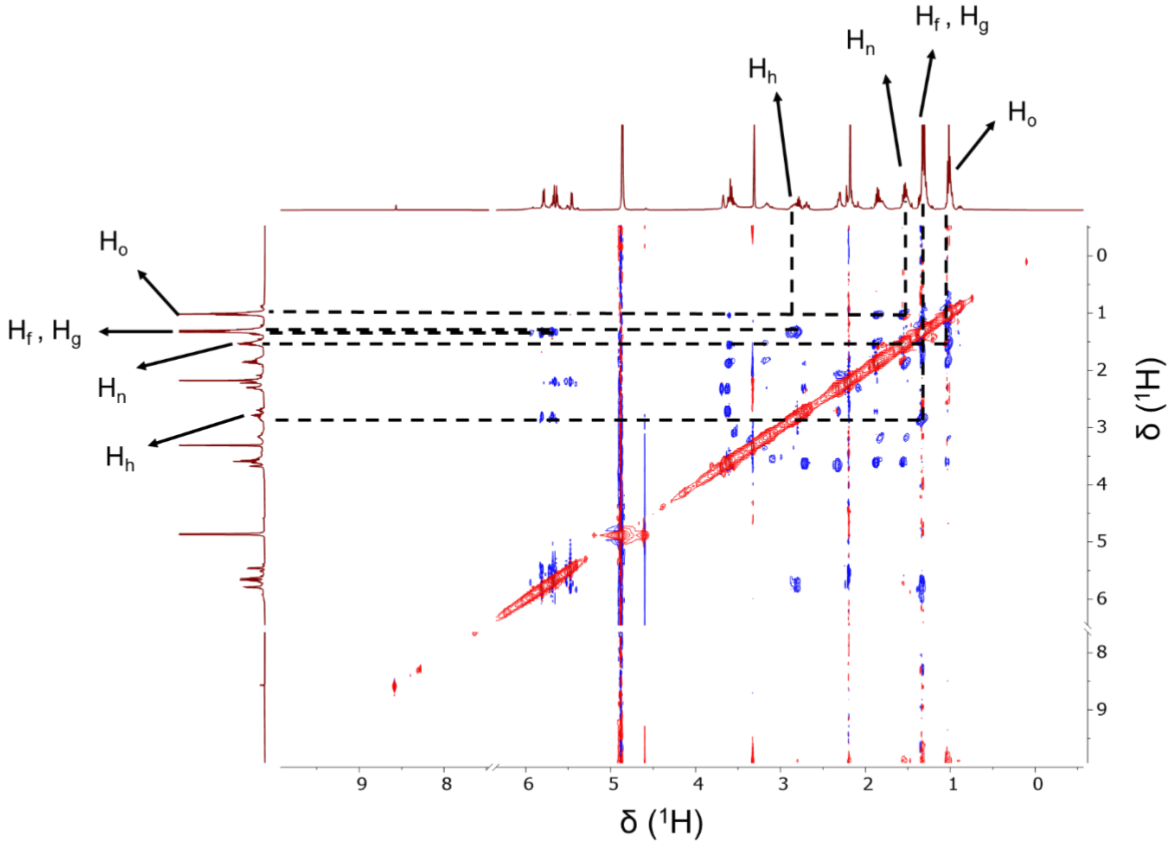

Figure S12. 500 MHz 2D ^{1}H-^{1}H NOESY NMR spectrum of Ru-LBSO recorded at 0.6 sec mixing time in methanol-d$_4$ showing H_n-H_o , H_h-H_f and H_h-H_g cross peak assignments.

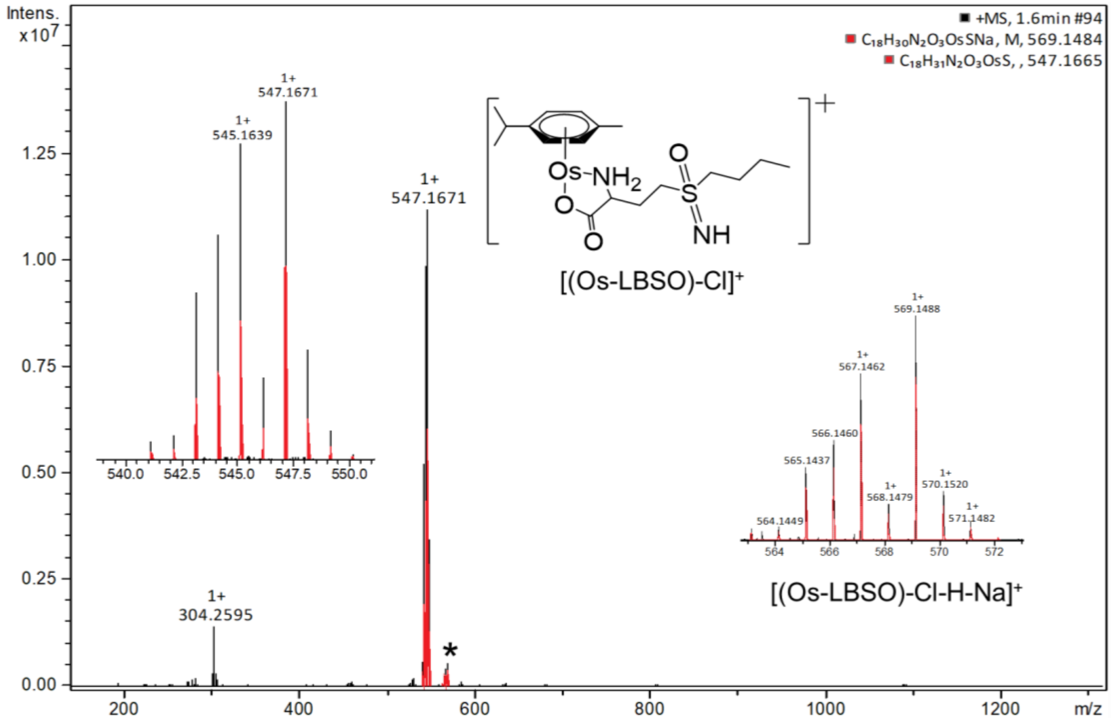

Figure S13. ESI-MS spectrum of Os-LBSO in MeOH. For the ion peak [(Os-LBSO)-Cl]$^{+}$ m/z calc. 547.1665 and m/z obs. 547.1761. * = sodium adduct [(Os-LBSO)-Cl-H+Na]$^{+}$ m/z calc. 569.1484 and m/z obs. 569.1488. As expected, both diastereomers give the same MS spectrum.

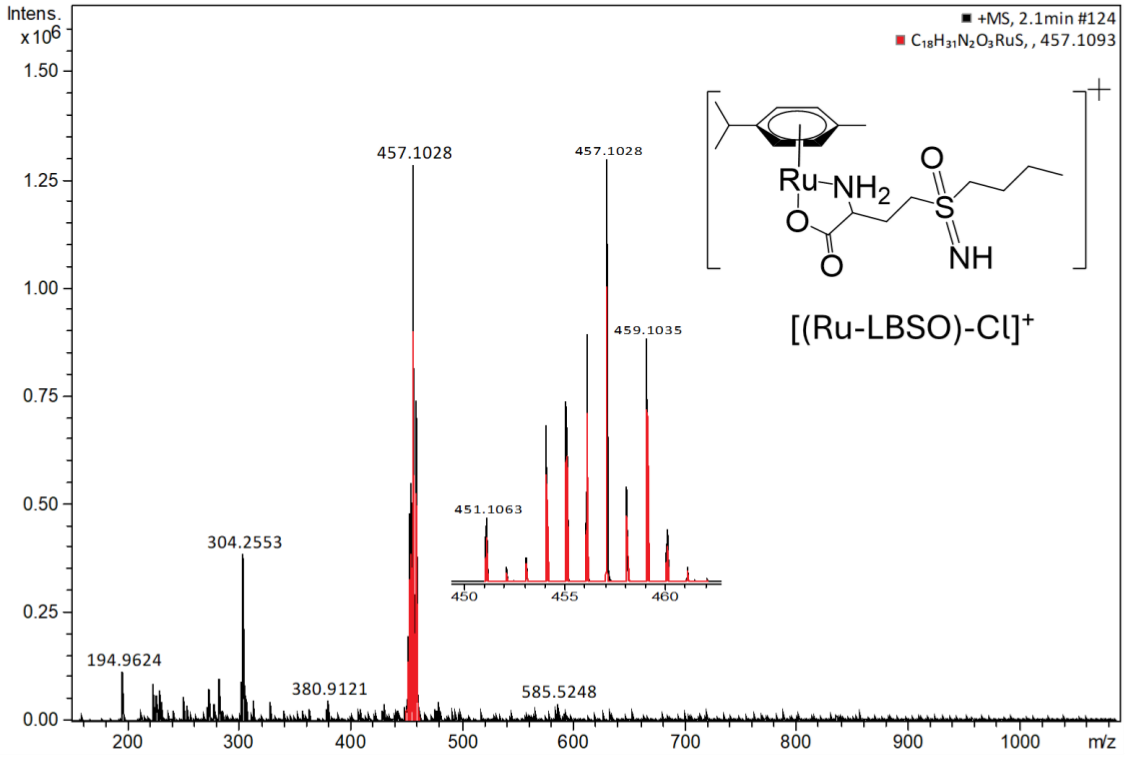

Figure S14. ESI-MS spectrum of Ru-LBSO in MeOH. For the ion peak [(Ru-LBSO)-Cl]$^{+}$ m/z calc. 457.1094 and m/z obs. 457.1028. As expected, both diastereomers give the same MS spectrum.

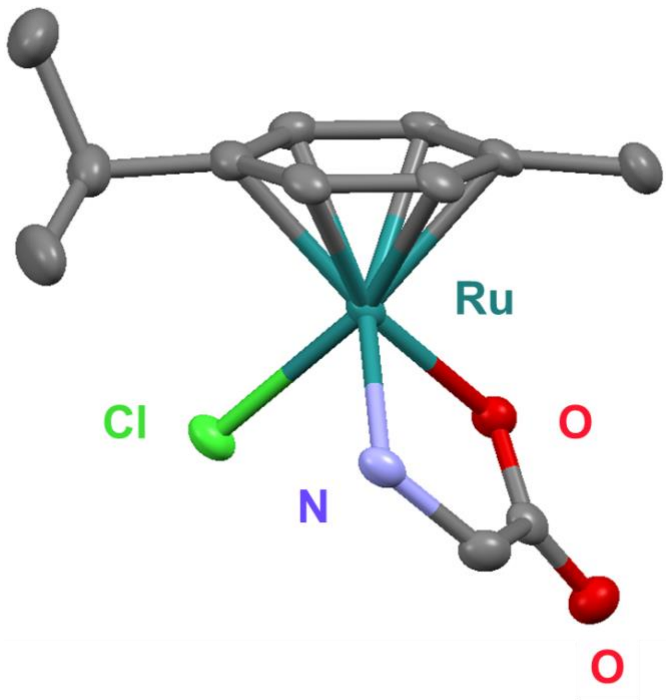

Figure S15. X-ray crystal structure of **Ru-Gly·CH_3OH** and thermal parameters drawn at 50% probability level. Hydrogen atoms and methanol solvent have been removed for clarity. The unit cell contains both Λ and Δ enantiomers and the compound is racemic.

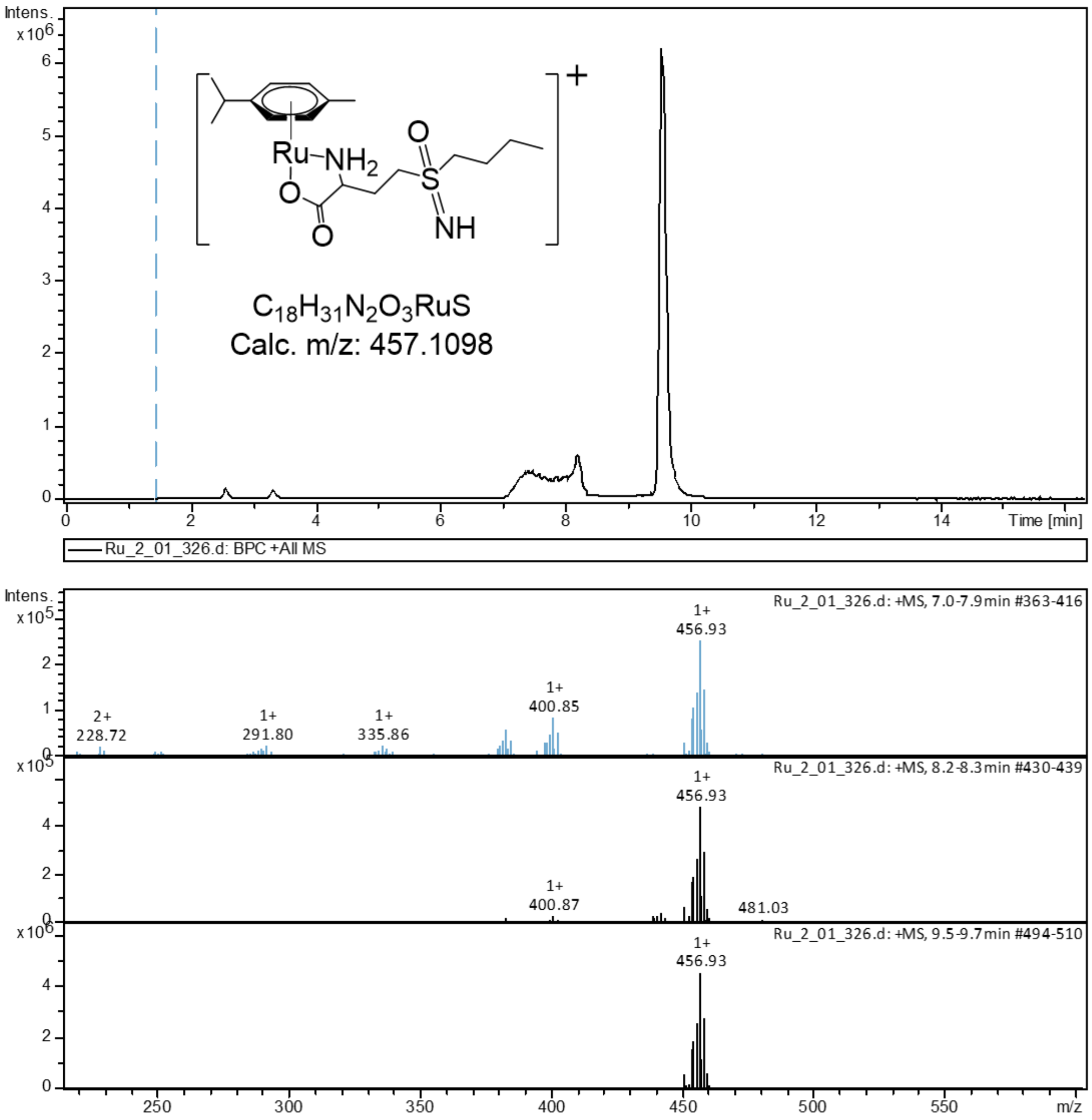

Figure S16. LC-MS of 100 µM Ru(II) L-BSO (acetonitrile : water , 0.1% formic acid). Same ion [(Ru-LBSO)-Cl]$^+$ is present in all three peaks. Sample was injected into the HPLC column Agilent ZORBAX Eclipse Plus C18 250 x 4.6 mm column with a pore size of 5 µm and analysed at a detection wavelength of 254 nm. Diastereomeric ratio is 70%:30%. Peaks at 7-8.3 min are presumed to be of a single diastereomer.

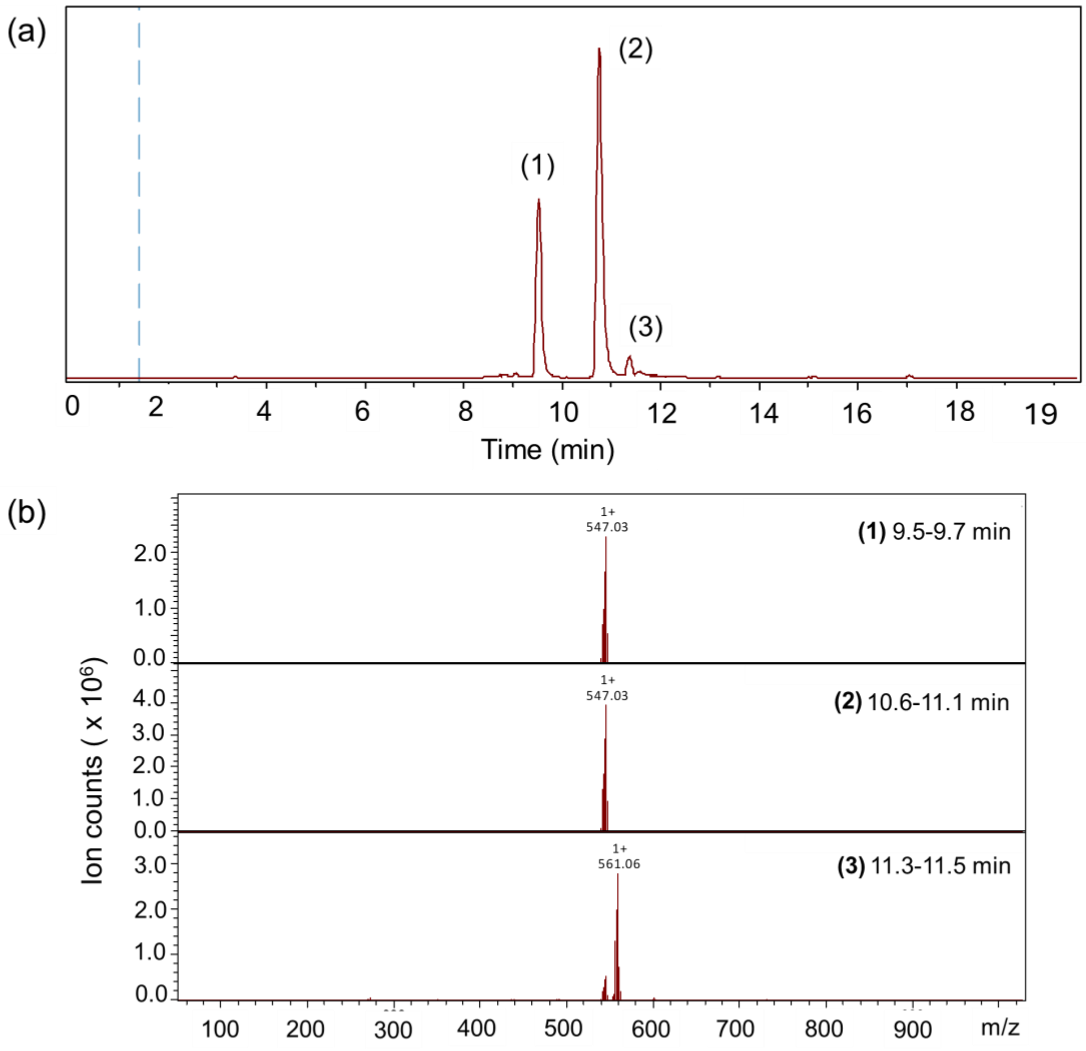

Figure S17. (a) HPLC of **Os-LBSO**. The sample (100 µM in 10% v/v acetonitrile-water 0.1% formic acid) was injected onto a C18 reverse-phase column with a detection wavelength of 254 nm. For elution gradient see Section S2.6. (b) MS of peaks 1-3 (ion counts) collected over the elution times indicated in (a). Peaks 1,2 with observed m/z 547.03 assignable to [(**Os-LBSO**)-Cl]$^{+}$ m/z 547.1 calculated for $C_{18}H_{31}N_2O_3OsS$. The minor peak eluting at 11.4 min, observed m/z 561.06, is unidentified.

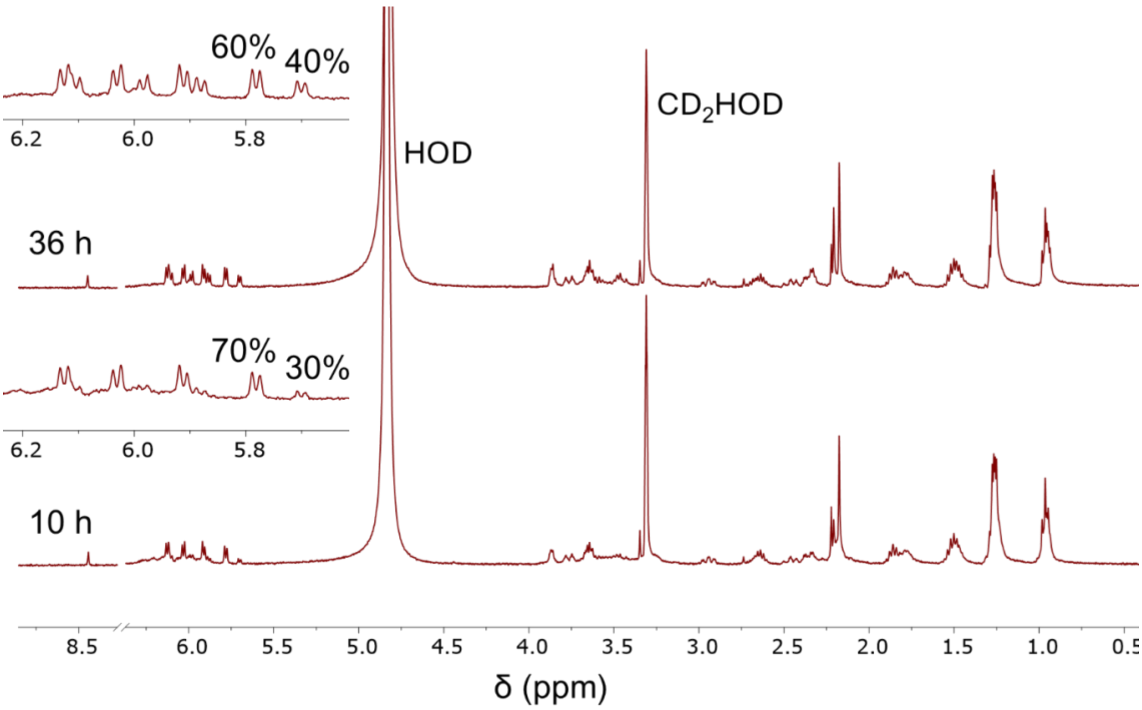

Figure S18. 400 MHz ^{1}H NMR spectra of Os-LBSO (2 mM) in 3:7 v/v methanol-d_4 : D_2O recorded 10 min and 36 h after dissolution at 310 K. Os-LBSO was completely hydrolysed within 15 min of sample preparation and reached an equilibrium with final diastereomeric ratio 60%:40%.

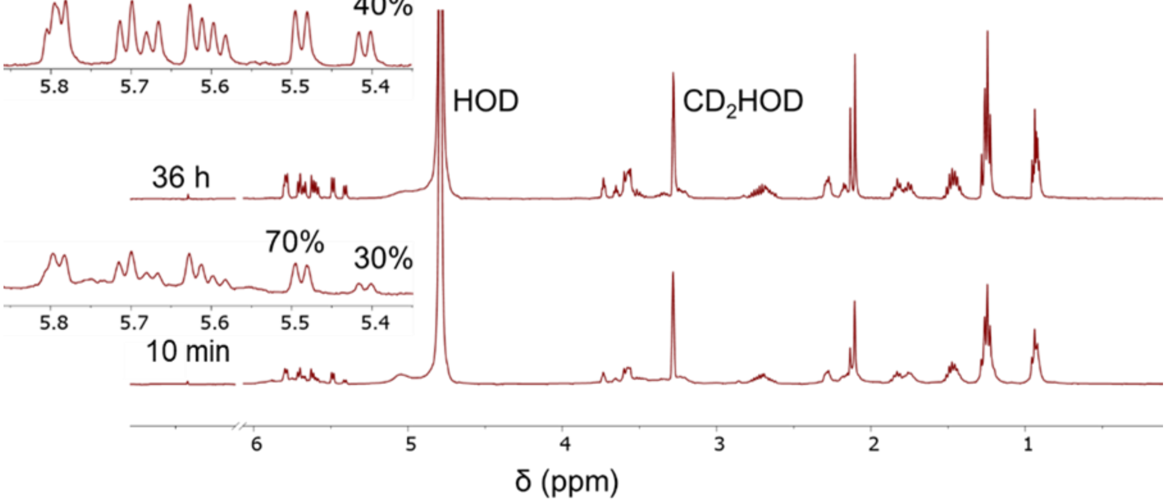

Figure S19. 400 MHz ^{1}H NMR spectra of Ru-LBSO (2 mM) in 3:7 v/v methanol-d_4 : D_2O recorded 10 min and 36 h after dissolution at 310 K. Ru-LBSO was completed hydrolysed within 15 min of sample preparation and reached an equilibrium with final diastereomeric ratio 60%:40%.

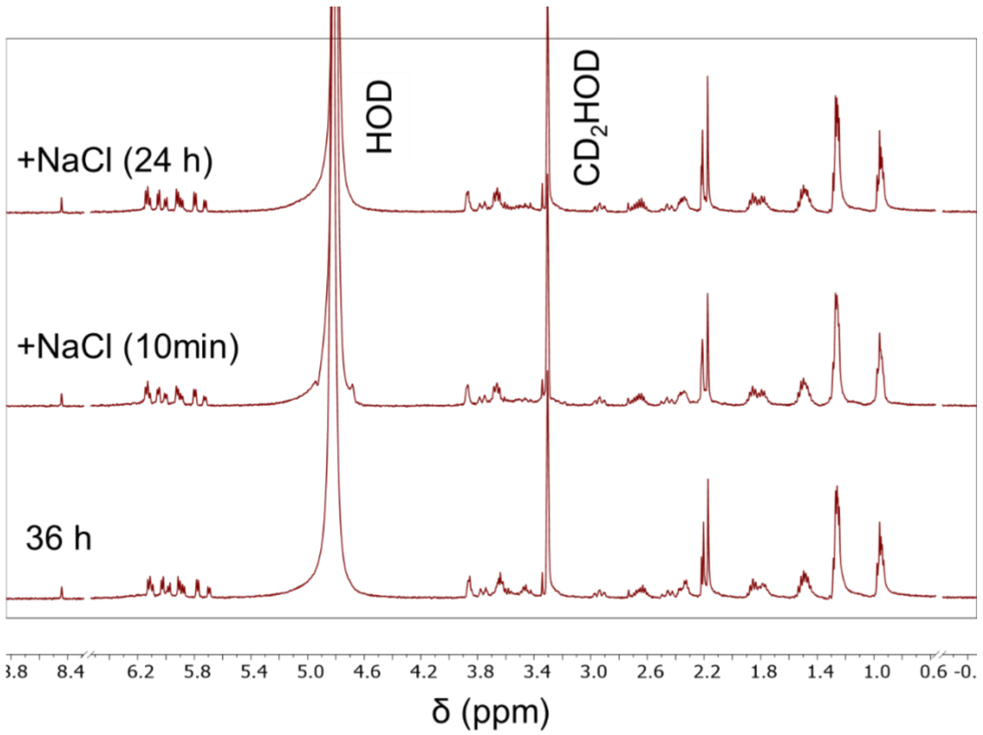

Figure S20. 400 MHz ^{1}H NMR spectrum of Os-LBSO after addition of NaCl (130 mM) to 36 h sample in 3:7 v/v methanol-d_4 : D_2O recorded after 10 min and 24 h. Reversal of hydrolysis was not observed.

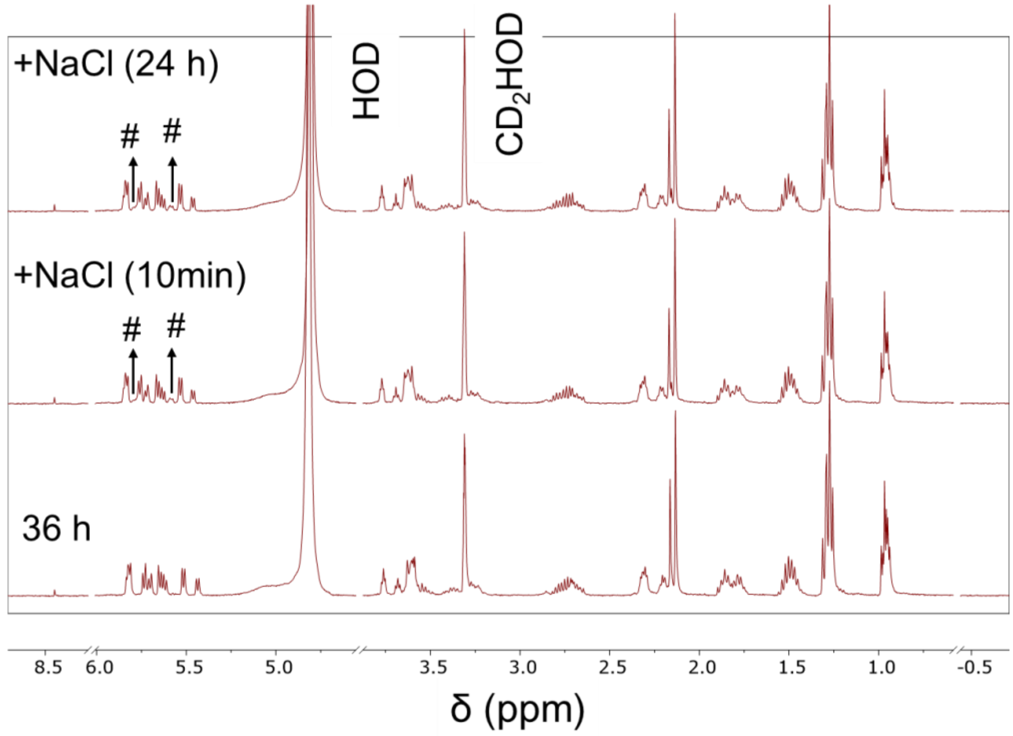

Figure S21. 400 MHz ^{1}H NMR of Ru-LBSO after addition of NaCl (130 mM) to 36 h sample in 3:7 v/v methanol-d_4 : D_2O recorded after 10 min and 24 h. Partial reversal of hydrolysis was observed, indicated with '#'.

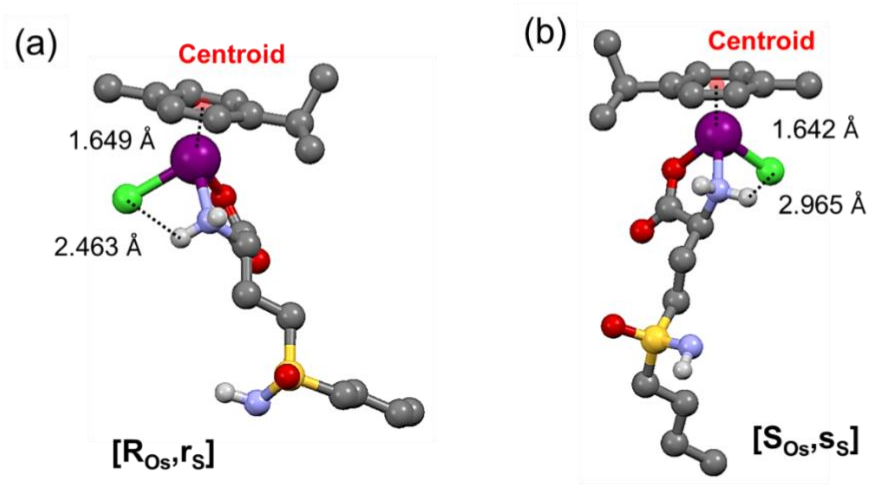

Figure S22. DFT optimised structures of diastereomers of Ru-LBSO. (a)[R_{Ru},r_S] (b)[S_{Ru},s_S] diastereoisomer of Ru-LBSO and the selected calculated interatomic distances Ru···Cl (2.428 Å), Ru···N (2.139 Å), Ru···O (2.074 Å), Ru···centroid (1.649 Å) and centroid···Cl (3.706 Å) for [R_{Ru},r_S]; Ru···Cl (2.428 Å), Ru···N (2.138 Å), Ru···O (2.076 Å), Ru···centroid (1.642 Å) and centroid···Cl (3.690 Å) for[S_{Ru},s_S].

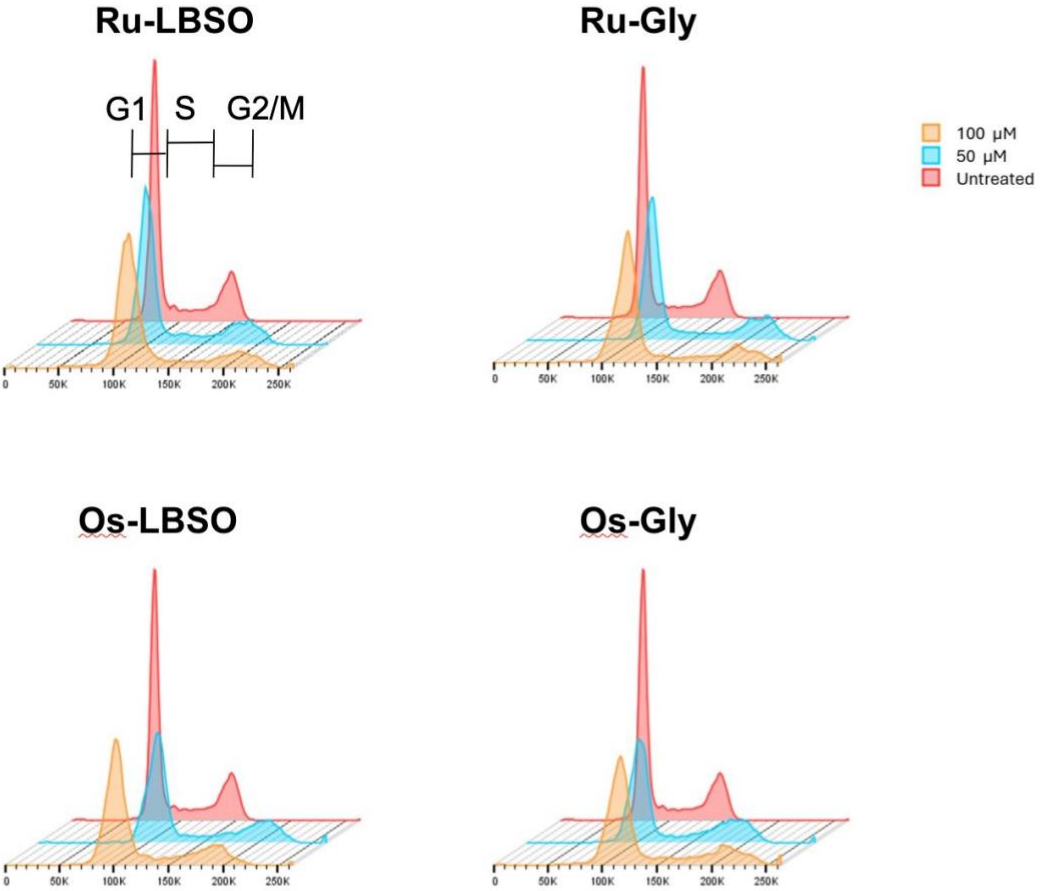

Figure S23. Representative flow cytometry plots showing the effect of Os/Ru L-BSO and glycine complexes at 50 or 100 µM on the phases of the cell cycle of IGROV-1 human ovarian cancer cells compared to untreated (control) cells. Percentage of cells in G1 (cell growth), S (DNA synthesis), G2/M, (mitosis) after 24 h treatment with complexes.

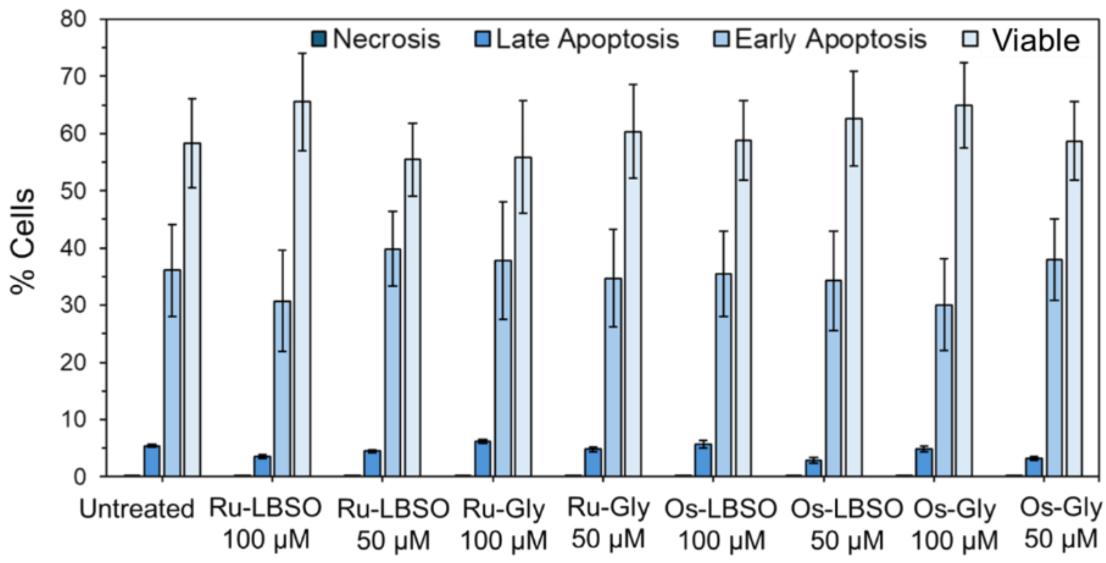

Figure S24. Effect of Os/Ru L-BSO and glycine complexes at 50 or 100 µM on the mode of death of IGROV-1 human ovarian cancer cells compared to untreated (control) cells. Percentage of IGROV-1 cancer cells in non-apoptotic, apoptotic (early and late) or necrotic phases determined by flow cytometry after 24 h treatment with Os/Ru L-BSO and glycine complexes. Neither necrosis nor apoptosis appear to be involved in the mechanism of cell death. Standard deviations calculated for N=3.

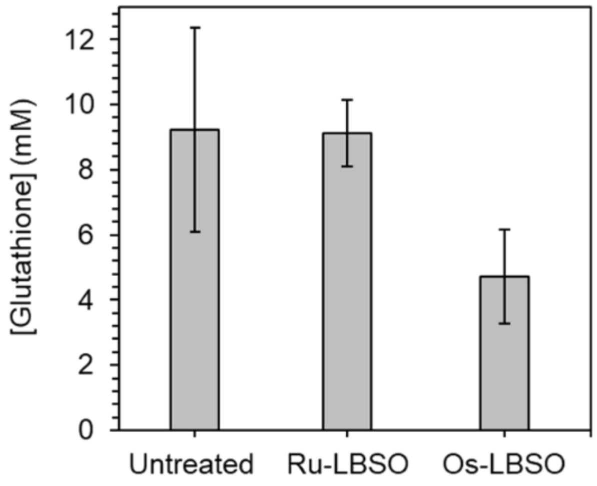

Figure S25. Effect of Ru/Os L-BSO at 50 µM complexes on the intracellular glutathione concentration in IGROV-1 human ovarian cancer cells after 24 h treatment.

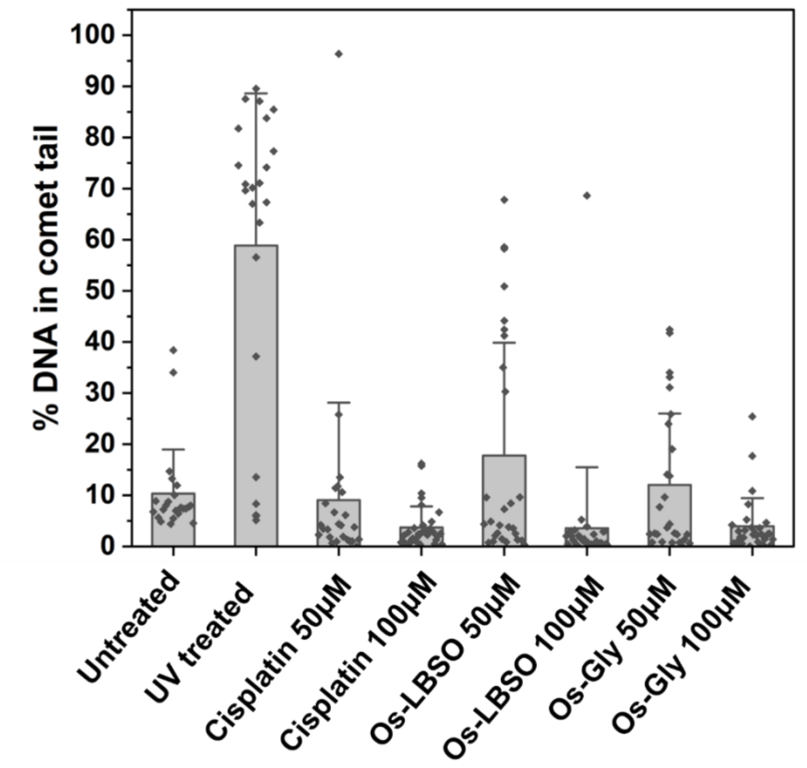

Figure S26. % DNA in comet tail for IGROV-1 cells following 24 h treatment with two osmium complexes Os-LBSO and Os-Gly. Approx. 20 comets per condition were measured. UV treated cells have statistically significant longer comet tails than untreated as expected.

3 References

(1) Bennett, M. A.; Huang, T. N.; Matheson, T. W.; Smith, A. K.; Ittel, S.; Nickerson, W., (η6-Hexamethylbenzene)Ruthenium Complexes. *Inorganic Syntheses* **1982**, 74-78.

(2) Habtemariam, A.; Melchart, M.; Fernandez, R. P.; Parsons, S.; D. H. Oswald, I.; Parkin, A.; P. A. Fabbiani, F.; E. Davidson, J.; Dawson, A.; E. Aird, R.; I. Jodrell, D.; Sadler, P. J. Structure−Activity Relationships for Cytotoxic Ruthenium(II) Arene Complexes Containing N,N-, N,O-, and O,O-Chelating Ligands. *J. Med. Chem.* **2006**, *49*, 6858–6868.

(3) Peacock, A. F. A.; Parsons, S.; Sadler, P. J. Tuning the Hydrolytic Aqueous Chemistry of Osmium Arene Complexes with N,O-Chelating Ligands to Achieve Cancer Cell Cytotoxicity. *J. Am. Chem. Soc.* **2007**, *129* (11), 3348–3357.

(4) Sheldrick, G. M. Crystal Structure Refinement WithSHELXL. *Acta. Crystallogr. C. Struct. Chem.* **2015**, A*71*, 3–8.

(5) Dolomanov, O. V.; Bourhis, L. J.; Gildea, R. J.; Howard, J. A. K.; Puschmann, H. OLEX2: A Complete Structure Solution, Refinement and Analysis Program. *J. Appl. Cryst.* **2009**, *42*, 339–341.

(6) Sheldrick, G. M. Crystal Structure Refinement WithSHELXL. *Acta. Crystallogr. C. Struct. Chem.* **2015**, C*71*, 3–8.

(7) Macrae, C. F.; Sovago, I.; Cottrell, S. J.; Galek, P. T. A.; McCabe, P.; Pidcock, E.; Platings, M.; Shields, G. P.; Stevens, J. S.; Towler, M.; Wood, P. A. Mercury 4.0: From Visualization to Analysis, Design and Prediction. *J. Appl. Crystallogr.* **2020**, *53*, 226–235.

(8) Vichai, V.; Kirtikara, K. Sulforhodamine B colorimetric assay for cytotoxicity screening. *Nat. Protoc.*, **2006**, *1*, 1112–1116.

(9) FlowJo™ Software (for Windows) Version 10. Ashland, OR: Becton, Dickinson and Company; 2023.

(10) Gyori, B. M.; Venkatachalam, G.; Thiagarajan, P. S.; Hsu, D.; Clement, M. V. OpenComet: an automated tool for comet assay image analysis. *Redox boil.*, **2014**, *2*, 457–465.

(11) Tao, J.; Perdew, J. P.; Staroverov, V. N.; Scuseria, G. E. Climbing the Density Functional Ladder: Nonempirical Meta–Generalized Gradient Approximation Designed for Molecules and Solids. *Phys. Rev. Lett.* **2003**, *91, 146401-146404*.

(12) a) Schäfer, A.; Horn, H.; Ahlrichs, R. Fully optimized contracted Gaussian basis sets for atoms Li to Kr. *J. Chem. Phys.*, **1992,** *97*, 2571-2577 b) Schäfer, A.; Horn, H.; Ahlrichs, R. Fully optimized contracted Gaussian basis sets of triple zeta valence quality for atoms Li to Kr. *J. Chem. Phys.*, **1994**, *100***,** 5829-5835.

(13) Grimme, S.; Antony, J.; Ehrlich, S.; Krieg, H. A consistent and accurate ab initio parametrization of density functional dispersion correction (DFT-D) for the 94 elements H-Pu Available to Purchase. *J. Chem. Phys.* **2010**, *132*, 154104.

(14) Weigend, F. Accurate Coulomb-fitting basis sets for H to Rn. *Phys. Chem. Chem. Phys*., **2006**, *8*, 1057-1065.

(15) Frisch, M.J., Trucks, G.W., Schlegel, H.B., Scuseria, G.E., Robb, M.A., Cheeseman, J.R., Scalmani, G., Barone, V., Petersson, G.A., Nakatsuji, H., Li, X., Caricato, M., Marenich, A.V., Bloino, J., Janesko, B.G., Gomperts, R., Mennucci, B., Hratchian, H. P., Ortiz, J.V., Izmaylov, A.F., Sonnenberg, J.L., Williams-Young, D., Ding, F., Lipparini, F., Egidi, F., Goings, J., Peng, B., Petrone, A., Henderson, T., Ranasinghe, D., Zakrzewski, V.G., Gao, J., Rega, N., Zheng, G.,Liang, W., Hada, M., Ehara, M., Toyota, K., Fukuda, R., Hasegawa, J., Ishida, M., Nakajima, T., Honda, Y., Kitao, O., Nakai, H., Vreven, T., Throssell, K., Montgomery Jr., J.A., Peralta, J.E., Ogliaro, F., Bearpark, M.J., Heyd, J.J., Brothers, E.N., Kudin, K.N., Staroverov, V.N., Keith, T.A., Kobayashi, R., Normand, J.; Raghavachari, K., Rendell, A.P., Burant, J.C., Iyengar, S.S., Tomasi, J., Cossi, M., Millam, J.M., Klene, M., Adamo, C., Cammi, R., Ochterski, J.W., Martin, R. L., Morokuma, K., Farkas, O., Foresman, J.B. and Fox, D.J. Gaussian 16, Revision A.03 , Gaussian, Inc., Wallingford CT, **2016**.
